# Single-cell multiomic analysis identifies macrophage subpopulations in promoting cardiac repair

**DOI:** 10.1172/JCI175297

**Published:** 2024-08-27

**Authors:** Mingzhu Fu, Shengtao Jia, Longhui Xu, Xin Li, Yufang Lv, Yulong Zhong, Shanshan Ai

**Affiliations:** 1 Guangdong Provincial Key Laboratory of Bone and Joint Degeneration Diseases, Department of Physiology, School of Basic Medical Sciences, Southern Medical University, Guangzhou, China.; 2 Department of Cardiology, Heart Center, Zhujiang Hospital, Southern Medical University, Guangzhou, China.

**Keywords:** Cardiology, Cardiovascular disease, Epigenetics, Macrophages

## Abstract

Cardiac mononuclear phagocytic cells (Cardiac MPCs) participate in maintaining homeostasis and orchestrating cardiac responses upon injury. However, the function of specific MPC subtypes and the related cell fate commitment mechanisms remain elusive in regenerative and nonregenerative hearts due to their cellular heterogeneities. Using spatiotemporal single-cell epigenomic analysis of cardiac MPCs in regenerative (P1) and nonregenerative (P10) mouse hearts after injury, we found that P1 hearts accumulate reparative *Arg1*^+^ macrophages, while proinflammatory *S100a9*^+^*Ly6c*^+^ monocytes are uniquely abundant during nonregenerative remodeling. Moreover, blocking chemokine CXCR2 to inhibit the specification of the *S100a9*^+^*Ly6c*^+^-biased inflammatory fate in P10 hearts resulted in elevated wound repair responses and marked improvements in cardiac function after injury. Single-cell RNA-Seq further confirmed an increased *Arg1*^+^ macrophage subpopulation after CXCR2 blockade, which was accomplished by increased expression of wound repair–related genes and reduced expression of proinflammatory genes. Collectively, our findings provide instructive insights into the molecular mechanisms underlying the function and fate specification of heterogeneous MPCs during cardiac repair and identify potential therapeutic targets for myocardial infarction.

## Abstract 2

Comparative single-cell multiomic analysis of macrophage subtypes in regenerative and nonregenerative hearts identifies pro-regenerative targets for heart repair after injury

## Graphical abstract

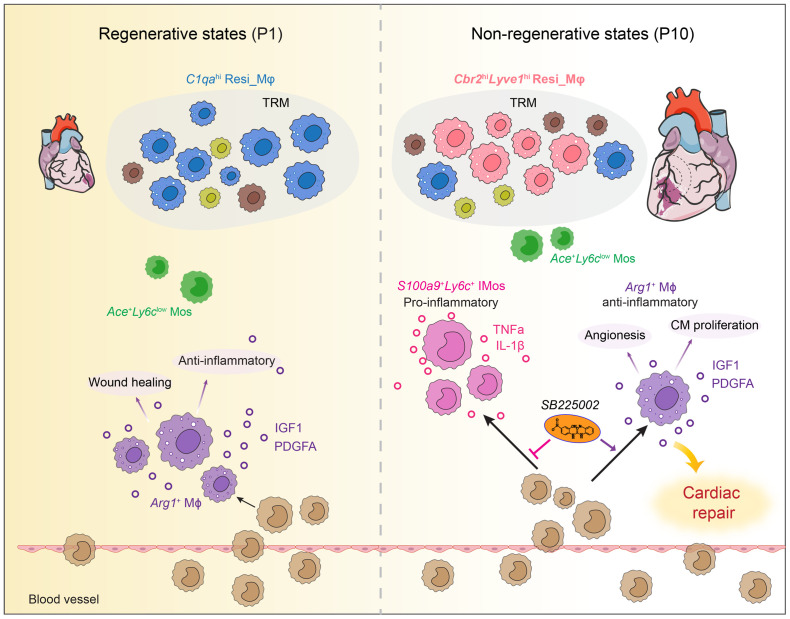

## Introduction

Cardiovascular disease is the primary cause of global mortality (1). Upon myocardial infarction (MI), the heart undergoes a diverse range of reparative processes, including the initiation of proinflammatory responses, followed by a transition toward an antiinflammatory state, ultimately culminating in wound healing. This finally leads to scar formation around the necrotic myocardium (2, 3). Macrophages, which constitute the major cardiac immune cell population, are capable not only of exacerbating heart damage but also of contributing to cardiac repair after MI (4). In the heart, the majority of resident macrophages are derived embryonically from either yolk sac macrophages or fetal liver monocytes and are perpetuated through self renewal. However, bone marrow monocyte-derived cardiac-resident macrophages infiltrate the heart around P14 and persist through continuous circulation (5).

Previous studies have shown that the regeneration potential of neonatal mouse hearts following MI is maintained until the first postnatal week of life (6). This regenerative paradigm affords an excellent model to investigate the molecular basis underlying the loss of spontaneous repair capacity in adult hearts. It has been demonstrated that macrophages exert a crucial role in neonatal mouse heart regeneration via paracrine effects (7). Specifically, neonatal hearts expand embryonic-derived resident macrophages, which exhibit functions in inflammation suppression, wound repair, and angiogenesis following MI (8). However, within injured adult hearts, the reparative tissue-resident macrophages are predominantly replaced by infiltrating inflammatory monocytes and monocyte-derived macrophages (9). These findings underscore the substantial potential of cardiac macrophages as promising therapeutic targets for addressing MI.

Interest in exploring the heterogeneity of cardiac macrophages in terms of identity, origin, and function upon MI has grown significantly. The advent of single-cell RNA-Seq (scRNA-Seq) has opened avenues to investigate the cellular diversity of cardiac macrophages at high resolution in an unbiased manner (10–14). However, most previous scRNA-Seq studies have primarily concentrated on scrutinizing macrophage functions in adult hearts after injury, and limited information is available regarding macrophage heterogeneity in neonatal hearts before the first week of life. Further, a systematic comparison of the differences in MPC composition and function in regenerative and nonregenerative hearts is lacking. Moreover, scRNA-Seq provides constrained insight into the regulatory mechanisms governing macrophage function specification. In contrast, single-cell chromatin profiling is capable of identifying cell-type-specific cis-regulatory elements, including enhancers, and can identify potential transcription factors exclusive to specific lineages. Consequently, the exploration of enhancer utilization dynamics upon cardiac injury has important implications for our understanding of the molecular basis underlying the fate and functional specification of macrophage subtypes. Nevertheless, the single-cell epigenomic landscape of cardiac macrophages remain undescribed.

In this context, we harnessed an epigenetic screen approach (CoBATCH (15) for H3K27ac) to survey the molecular regulators and prospective lineage of cardiac mononuclear phagocytic cells (MPCs) in P1 and P10 mouse hearts after MI. Leveraging this atlas, we identified cell-type–specific enhancer networks of relevance to cardiovascular disease traits and identified transcription factors instrumental in the establishment of individual cell identities. Notably, we uncovered a monocyte-derived reparative *Arg1*^+^ macrophage subtype that exhibited higher enrichment in P1 hearts after injury, while a monocyte-derived proinflammatory monocyte subpopulation characterized by *S100a9* and *Ly6c* expression was uniquely abundant in P10 hearts 3 days after MI. Pharmacological targeting of the CXCR2 chemokine to block the generation of *S100a9*^+^*Ly6c*^+^ proinflammatory monocytes in P10 hearts led to substantial improvement in cardiac function after MI. Mechanistically, as demonstrated by our scRNA-Seq analysis, CXCR2 inhibition increased the number of *Arg1*^+^ macrophages with increased expression of wound repair–related genes and reduced expression of proinflammatory genes, which resulted in increased proliferation of cardiomyocytes and endothelial cells, along with increased survival of cardiomyocytes during cardiac repair. Moreover, the *Arg1*^+^ and *S100a9*^+^*Ly6c*^+^ subpopulations were also identified in adult hearts after MI, exhibiting the weakest wound healing and strongest proinflammatory activities, respectively. Taken together, our findings underscore the potency of single-cell multiomic analysis in elucidating the function and cell fate commitment mechanisms of MPC subtypes upon cardiac injury and highlight the potential therapeutic strategy of targeting *S100a9*^+^*Ly6c*^+^ infiltrating monocytes for heart failure.

## Results

### Regional composition dynamics of cardiac MPCs between regenerative and nonregenerative mouse hearts after injury.

To obtain a comprehensive appreciation of the temporally and spatially resolved dynamics of cardiac MPCs in infarcted hearts with different regenerative potential, we performed flow cytometry analysis on cardiac CD45^+^F4/80^+^ MPCs derived from sham and MI hearts at 3 and 7 days after P1 (regenerative) (P1-MI_3D and P1-MI_7D) and P10 (nonregenerative) (P10-MI_3D and P10-MI_7D) injury. Consistent with the regional differences, we observed disparities in the spatial compositions of CD45^+^F4/80^+^ MPCs in the infarct zone (IZ), border zone (BZ), and remote zone (RZ) (Supplemental Figure 1, A–C; supplemental material available online with this article; https://doi.org/10.1172/JCI175297DS1). A dramatic increase in the number of macrophages in the IZ was observed 3 days after MI in both P1 and P10 hearts, but the number of macrophages in the IZ gradually decreased during pathological progression (Supplemental Figure 1, B and C). Increased accumulation of macrophages was noticeable in both the BZ and RZ of P10-MI_7D hearts compared with P1-MI_7D hearts, a state that might contribute to the exacerbated inflammatory responses and unfavorable cardiac remodeling in P10 hearts after injury (16). Additional immunofluorescence staining for F4/80 in cardiac tissue sections confirmed these observations (Supplemental Figure 1, D and E). In conclusion, cardiac MPCs exhibited distinct kinetics of regional composition in regenerative and nonregenerative hearts, inspiring us to further explore the molecular basis underlying this observation.

### Single-cell CoBATCH profiling of cardiac MPCs at spatiotemporal resolution in regenerative and nonregenerative mouse hearts.

**Figure 1:**
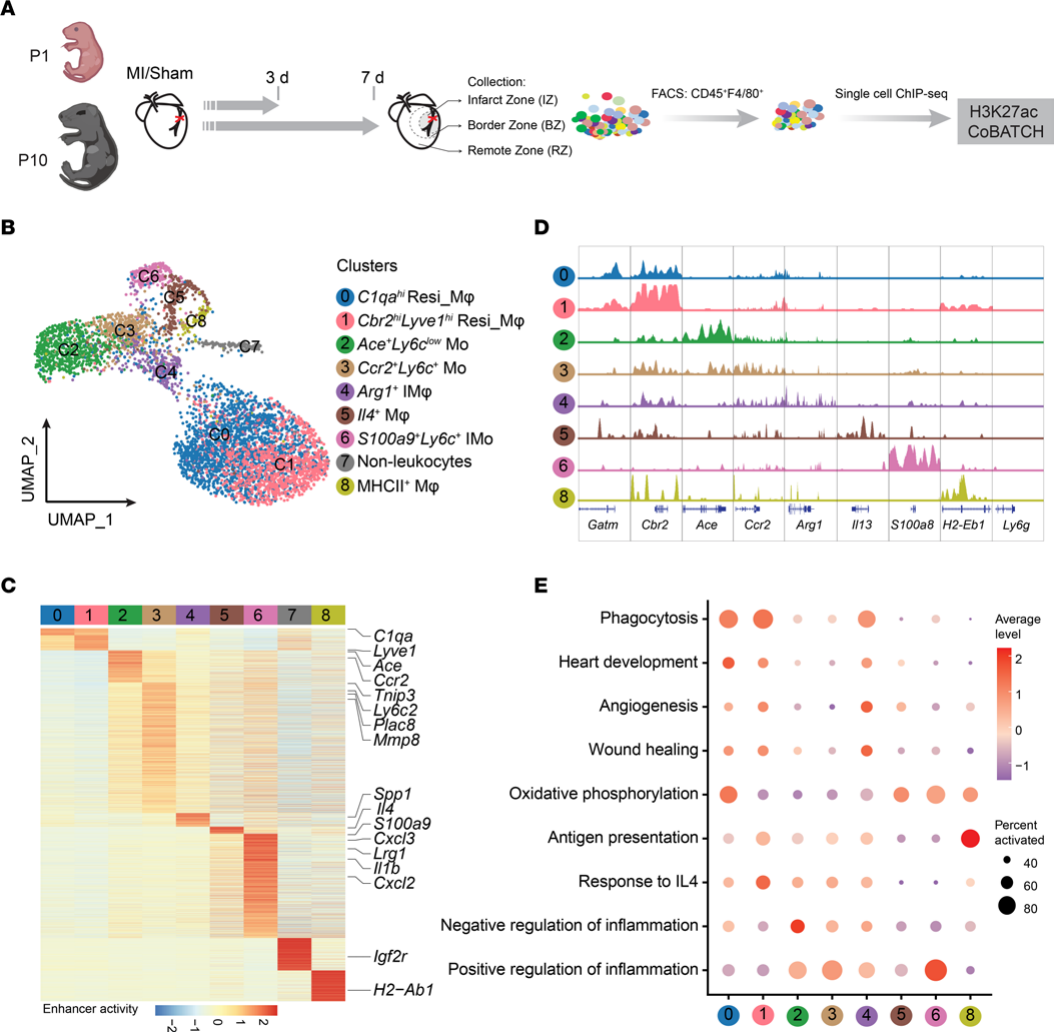
Single-cell H3K27ac ChIP-Seq of MPCs in hearts at 3 and 7 days after P1 and P10 MI/sham. (A) Schematic representation of the experimental design. (B) UMAP plot of 5,226 cardiac CD45^+^F4/80^+^ MPCs identified 9 different clusters. (C) Heatmap showing normalized cell-type specific H3K27ac ChIP-Seq signals and representative nearby genes were labeled for each subcluster. (D) Genome browser view of H3K27ac signals around cluster-specific marker genes. (E) Dot plot showing the enhancer score of nearby genes participating in typical functions among all clusters.

Enhancers, which are cis-regulatory DNA elements, play a crucial role in shaping gene expression patterns and specifying cell fate (17). To investigate the kinetics of enhancer usage in controlling macrophage dynamics in hearts with distinct regeneration potential, we performed time-resolved single-cell CoBATCH, a recently developed single-cell ChIP-Seq method (15), to examine H3K27ac modifications, which mark active enhancers (18), in CD45^+^F4/80^+^7AAD^–^ MPCs isolated 3 and 7 days after MI in P1 and P10 hearts (Figure 1A). A total of 2,594 and 2,632 cells originating from MI and sham hearts, respectively, met the stringent selection criteria, including nonduplicated reads per cell (> 1,500) and percentage of reads in peaks (> 10%) (Supplemental Table 1), yielding an average of 7,290 unique reads per cell (Supplemental Figure 2A). Subsequently, the merged 54,045 H3K27ac peaks derived from 24 aggregated bulk samples were employed to define active enhancer regions. A single-cell binary matrix of H3K27ac ChIP-Seq signals within the 54,045 peaks was then constructed (Supplemental Table 2). The Seurat package was further utilized to delineate the cellular composition, and the batch correction was carried out using “Harmony” for the 2 biological replicates (19) (Supplemental Figure 2B).

Based on the resolution cluster tree analysis, a total of 9 subclusters were singled out within the CD45^+^F4/80^+^ MPC population, along with enhancer peaks specific to each cluster (Figure 1, B and C, Supplemental Figure 2C, and Supplemental Table 3). To annotate the resulting cell clusters, we used enhancer signals spanning from 50 kb upstream to 30 kb downstream of the gene body as a proxy for gene activity. Cluster C7 was excluded from further analysis because it consisted of nonleukocytes displaying higher enhancer activities at loci such as *Igfbp7*, *Col1a1,* and *Col4a1* and lacking *Adgre1* enhancer signals (Supplemental Figure 2, D and E). C0 and C1, which are the primary macrophage clusters, exhibited elevated enhancer activities surrounding markers for tissue-resident macrophages, such as *Lyve1*, *C1qa*, *Mgl2*, and *Fcrls* (12, 13, 20, 21) (Figure 1C and Supplemental Figure 2, D and E). C1 corresponded to *Cbr2*^hi^*Lyve1*^hi^ cardiac macrophages (Mφ) (5), while cluster C0 was assigned to the *C1qa^hi^* Resi_Mφ population, which decreased strikingly after MI (Figure 1, C and D, and Supplemental Figure 2, D-F). Gene Ontology enrichment analysis revealed that C0 and C1 were primarily engaged in processes such as phagocytosis, heart development, and antigen presentation (Figure 1E, Supplemental Figure 2G, and Supplemental Table 4).

Cluster C3 exhibited the highest enhancer activities around *Ly6c2* and *Ccr2* and was thus defined as classical *Ccr2*^hi^*Ly6c*^hi^ monocytes (Figure 1, C and D, and Supplemental Figure 2, D, E, J and K). Moreover, C2 displayed significant enhancer enrichment adjacent to *Ace* and *Nr4a1* but relatively lower enrichment for *Ly6c2*, corresponding to nonclassical *Ly6C*^lo^ monocytes (22) (Figure 1, C and D, and Supplemental Figure 2, D and E). Functional analysis of feature genes near cluster-specific enhancers revealed that classical monocytes C3 participated in proinflammatory reactions, while *Ly6c*^lo^ nonclassical monocytes C2 were primarily involved in antiinflammatory processes (23) (Figure 1E and Supplemental Figure 2G). Additionally, the cells in cluster C8 resembled MHCII^+^ cardiac-resident macrophages (13), as enhancer activities related to antigen presentation genes (*H2-Aa*, *H2-Eb1*, *H2-Ab1*) were notably enriched in this cluster (24) (Figure 1, C-E, and Supplemental Figure 2, D and E). Unexpectedly, we identified an *Il4^+^* macrophage cluster (C5) that exhibited the highest gene activity around the *Il4* loci (Figure 1B and Supplemental Figure 2, D and E), and this cluster was not reported in previous scRNA-Seq studies. The discrepancy could potentially be caused by the differences in gene activity based on the surrounding enhancer signals and gene expression level defined by the copy number of its transcripts, reflecting the distinctive level of cellular heterogeneity revealed through single-cell transcriptional and epigenomic profiling.

**Figure 2:**
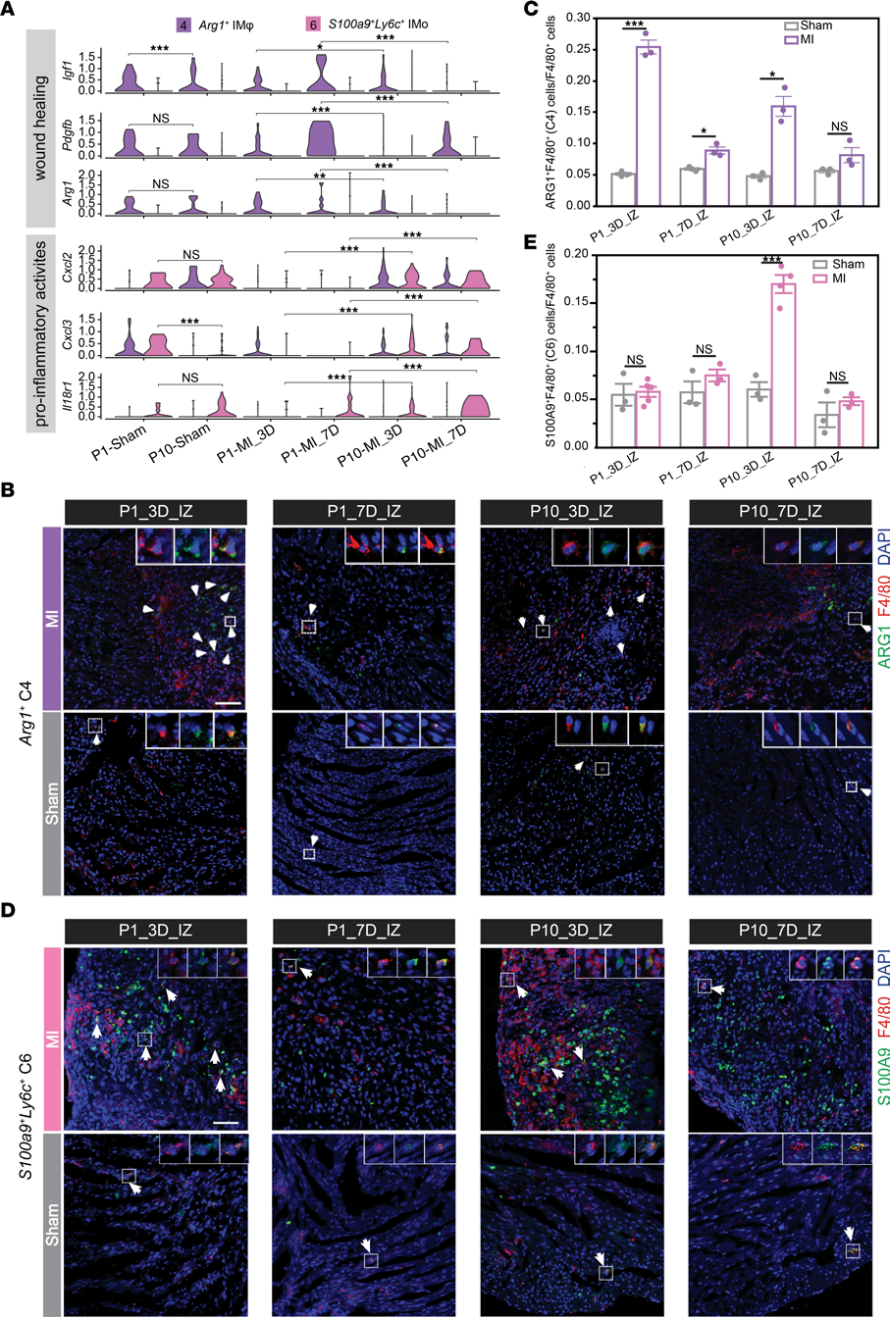
Function and abundance characterization of *Arg1*^+^ C4 and *S100a9*^+^*Ly6c*^+^ C6 cells in neonatal myocardial infarcted hearts. (**A**) Violin plots showing the enhancer activities of representative genes participated in wound healing and proinflammatory activities among *Arg1*^+^ C4 and *S100a9*^+^*Ly6c*^+^ C6. (**B** and **C**) Representative immunostaining (**B**) and quantification (**C**) for F4/80^+^ARG1^+^ C4 cells in the IZ of mouse hearts 3 and 7 days after P1 and P10 MI/sham. Scale bars: 50 μM. (**D** and **E**) Representative immunostaining (**D**) and quantification (**E**) for F4/80^+^S100A9^+^ C6 cells in the IZ of mouse hearts at 3 and 7 days after P1 and P10 MI/sham. Scale bars: 50 μM. *n* = 3–5 mice per experimental group. Data represent mean ± SEM. The *P* value was determined by Kruskal-Wallis *H* test, followed by Dunn’s test in **A**, and unpaired 2-tailed Student’s *t* test in (**C** and **E**). *P < 0.05; **P < 0.01; ***P < 0.001.

Following MI, the generation of cells in clusters C4 and C6 was significantly induced (Supplemental Figure 2F). C4 exhibited higher enhancer activities for *Arg1* (9), *Trem2* (12), the lipid-associated macrophage (LAM) marker genes *Gpnmb* and *Spp1* (25), and the wound repair gene *Igf1* (26), resembling the *Trem2*^hi^*Spp1*^hi^ cluster identified by Rizzo et al. (10) (Figure 1, C and D, and Supplemental Figure 2, D, E and H). Accordingly, *Arg1*^+^ C4 was predominantly engaged in wound healing and angiogenesis processes (Figure 1E and Supplemental Figure 2G), exhibiting higher enhancer activity around wound repair genes such as *Igf1*, *Pdgfb*, and *Arg1* 3 and 7 days after P1 injury than after P10 injury (27, 28) (Figure 2A). Interestingly, cluster C6, infiltrated monocytes (IMos), displayed positive enhancer signals around not only *S100a9*/*S100a8* and *Ly6c2* (Figure 1, C and D, and Supplemental Figure 2, D and E) but also granulocyte-associated genes such as *Lcn2*, *Cd177*, and *Wfdc21* (29) (Supplemental Figure 2I). Moreover, the majority of *S100a9*^+^*Ly6c*^+^ C6 cells were negative for Ly6G (Supplemental Figure 3, A-C) and displayed typical mononuclear morphology, thus resembling monocytes or macrophages rather than neutrophils (Supplemental Figure 3D). In line with its strong proinflammatory enrichment (Figure 1E and Supplemental Figure 2G), *S100a9*^+^*Ly6c*^+^ C6 featured higher enhancer enrichment near genes such as *Cxcl2*, *Cxcl3*, and *Il18r1* at 3- and 7-days after MI in P10 than in P1 hearts (Figure 2A).

Consistent with the distinct regenerative capacities of P1 and P10 hearts, we observed distinct patterns in the preferences for regional accumulation of the proreparative *Arg1*^+^ C4 cluster and the proinflammatory *S100a9*^+^*Ly6c*^+^ C6 cluster 3 days after MI in P1 and P10 hearts (Supplemental Figure 4A). Specifically, the generation of *Arg1*^+^ C4 and *S100a9*^+^*Ly6c*^+^ C6 cells was significantly induced at the IZ of P1-MI_3D and P10-MI_3D hearts, respectively (Figure 2, B–E and Supplemental Figure 4, B and C). Moreover, *S100a9*^+^*Ly6c*^+^ C6 displayed enrichment not only in the IZ but also in BZ regions of P10-MI_3D hearts, reflecting more pronounced inflammatory responses in P10-MI_3D hearts than in P1-MI_3D hearts (Figure 2, D and E and Supplemental Figure 4, A–G). While *Arg1*^+^ C4 was also induced in P10 heart following MI, its abundance was comparatively lower than that observed in P1 hearts (Figure 2, B and C and Supplemental Figure 4, H and I). Consequently, the prompt reparative responses in P1 hearts, along with more severe inflammatory response in P10 hearts after MI, may underlie the discernible differences in regeneration potential between P1 and P10 mouse hearts.

Collectively, these findings underscore the capability of our high-quality single-cell H3K27ac CoBATCH dataset to identify distinct MPC subtypes and delineate the functional heterogeneity of cardiac MPCs in both regenerative and nonregenerative hearts.

### Decoding cluster-specific enhancer regulatory networks of cardiac MPCs.

**Figure 3:**
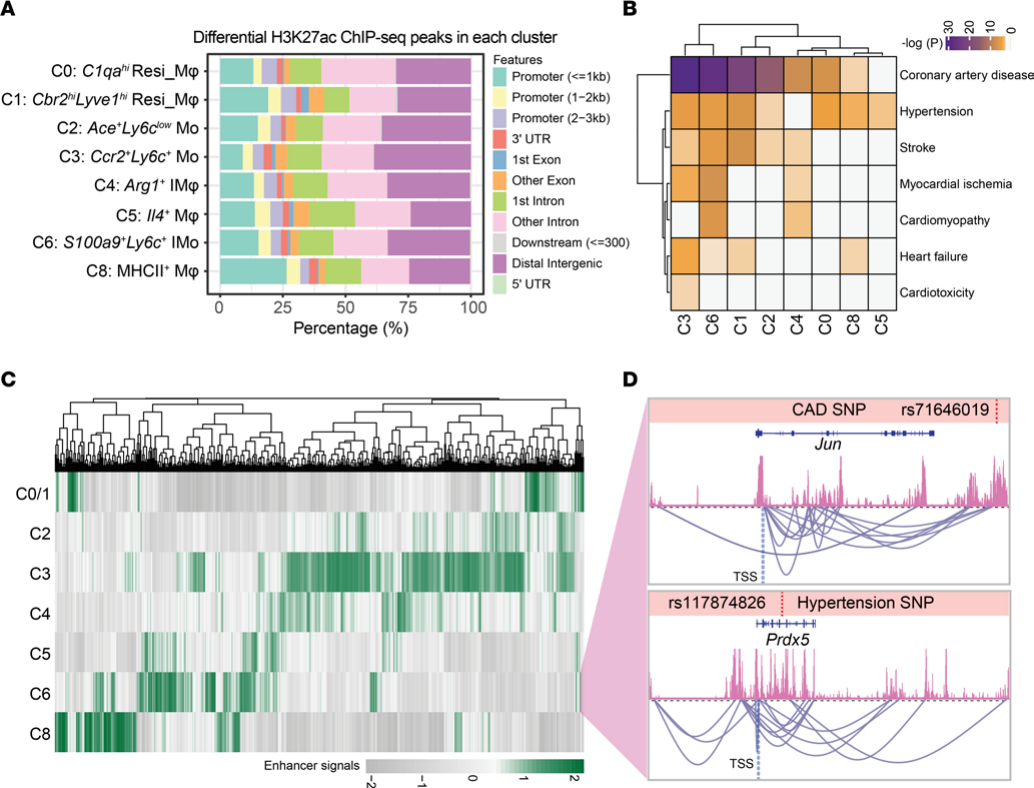
Enrichment analysis of GWAS signals for cardiovascular disease traits within cell-type–specific enhancers. (**A**) Bar plot of annotated genomic features of cluster-specific H3K27ac peaks (fold change > 2). (**B**) Heatmap showing enrichment of GWAS SNPs associated with cardiovascular disease traits in cell-type–resolved enhancers. (**C**) Heatmap showing normalized H3K27ac ChIP-Seq signals within peaks among the 14,977 cis-correlation networks. (**D**) Track viewer showing the aggregated H3K27ac ChIP-Seq signals of *S100a9*^+^*Ly6c*^+^ C6 along with cell-type specific cis-correlation networks centered around the *Jun* and *Prdx5* loci. The cis-correlations between peaks were shown by carmine arches and the SNPs for CAD (coronary artery disease) and hypertension were highlighted by red dashed lines.

Given that a significant fraction of genome-wide association study–identified (GWAS-identified) common human variants are located in cell-type specific distal regulatory elements (30), we were interested in exploring the enrichment of genetic variants associated with cardiovascular diseases in each subpopulation. To this end, Seurat (Log_2_FC > 0.25, *P_adj_* < 0.05) was first utilized to identify enhancer peaks unique to each cluster. As expected, the majority of cell-type specific peaks were situated in intronic and distal enhancer regions, consistent with previous studies indicating the high cell-type specificity of distal enhancers (18) (Figure 3A). Next, we extracted single-nucleotide polymorphisms (SNPs) associated with cardiovascular diseases from the GWAS database (31) and accurately lifted over to the orthologous mouse genome, resulting in the identification of 4,313 SNPs (Supplemental Table 5). Using the binomial test, we examined the enrichment of trait-associated variants within cell type–specific enhancers. Our analysis revealed that inflammatory monocytes (C3 and C6) exhibited the highest enrichment for various cardiovascular disease–associated SNPs (Figure 3B and Supplemental Table 6). This finding emphasizes the significant role of monocyte-mediated inflammatory reactions in cardiovascular diseases (32), particularly in the context of coronary artery diseases (CAD) (33).

Noncoding genetic variants enriched within enhancers are believed to have the ability to modulate the expression level of target genes (34). Therefore, we employed Cicero to probe the interactions among cluster-specific enhancers identified by H3K27ac ChIP-Seq signals (35). A total of 14,977 cis-correlation networks (CCRNs) were identified among all single cells, with 14,303 CCRNs displaying cell-type specificity (Figure 3C and Supplemental Table 7). For instance, the proinflammatory genes *Jun* (36) and *Prdx5* (37) exhibited the highest representation in CCRNs specifically in the *S100a9*^+^*Ly6c*^+^ C6 cluster, in which the SNPs associated with CAD and hypertension were also notably enriched (Figure 3D and Supplemental Figure 5, A and B).

**Figure 4:**
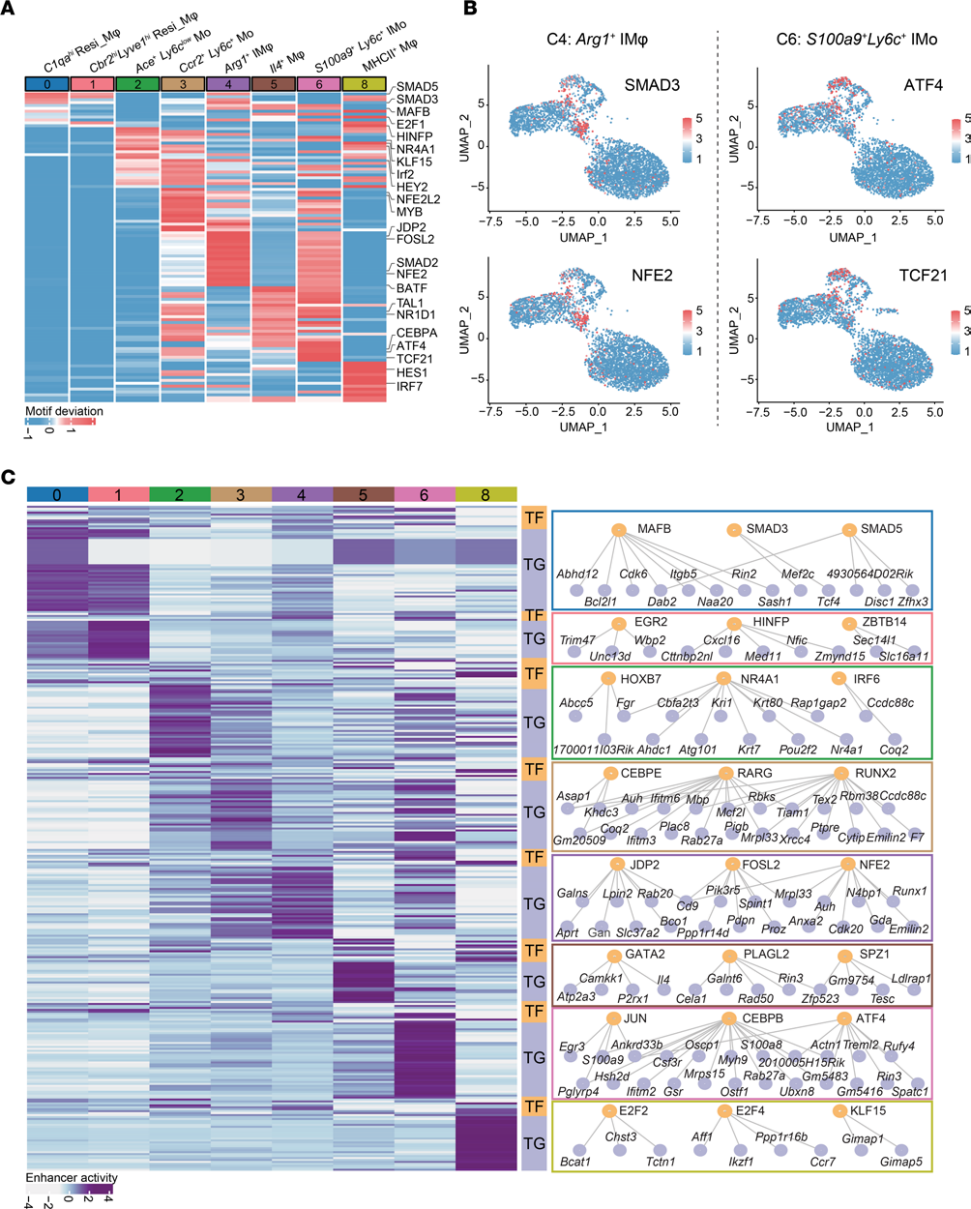
Characterization of cell-type–specific TF regulatory networks. (**A**) Heatmap showing the average ChromVAR motif activities of 107 most variable TFs across each cluster. The color bar represents values normalized by *z* score for each row. (**B**) Feature plots showing the representative cluster-specific ChromVAR motif activities of SMAD3 and NFE2 in C4, as well as ATF4 and TCF21 in C6. (**C**) Enhancer activities of critical TFs and their target genes (TG) in each cluster. The orange nodes indicate TFs and the violet nodes indicate corresponding target genes (TG) in the representative networks on the right panel. The color bar represents values normalized by *z* score for each row.

Transcription factors (TFs) have been demonstrated to be pivotal determinants in shaping the fate and functional specifics of cardiac macrophages (38). Therefore, we interrogated the shared and unique regulatory TFs across different clusters by applying ChromVAR (39) (Figure 4A and Supplemental Table 8). For example, *Arg1*^+^ C4 exhibited the highest motif activity for SMAD3, coincident with the phagocytic and antiinflammatory characteristics of these cells (40) (Figure 4, A and B). Additionally, the antiinflammatory transcription factor NFE2 was markedly enriched in *Arg1*^+^ C4 (Figure 4, A and B), underscoring its potential involvement in shaping the functionality of this particular cluster. Notably, we observed specific enrichment of the TF NR4A1 in C2, which has been linked to the specification of the *Ly6c*^lo^ monocyte lineage (41). Conversely, the motif activities of the proinflammatory TFs ATF4 and TCF21 were specifically enriched in *S100a9*^+^*Ly6c*^+^ C6 (42, 43) (Figure 4, A and B). Additionally, we identified enrichment of the antigen presentation regulator IRF7 in C8 (44) (Figure 4A). These findings elucidated the known and potentially novel TFs that contribute to the functional specification of each distinct cell subtype.

To further explore the functional TFs within each cluster, we analyzed the TF downstream target gene (TG) networks among all clusters based on the rationality that cell type–critical TFs should not only exhibit specific enrichment in each cluster but also actively participate in gene expression regulation. Leveraging the TF-TG database from CellNET (45), we identified 70 crucial TFs among 8 subpopulations and subsequently characterized cluster-specific TF-TG networks (Figure 4C and Supplemental Table 9). For example, genes such as *Egr3* (46), *S100a9*, *S100a8* (47), and *Treml2* (48), which are involved in inflammatory responses, were regulated by critical TFs, such as JUN, CEBPB, and ATF4, in *S100a9*^+^*Ly6c*^+^ C6 (Figure 4C). In summary, our single-cell H3K27ac ChIP-Seq data not only facilitated the identification of cell type–specific enhancers harboring cardiovascular disease–associated SNPs but also enabled us to explore the critical TFs essential for establishing cluster-specific functions and identities.

### Comparative analysis of the epigenetic features between P1 and P10 cells.

**Figure 5:**
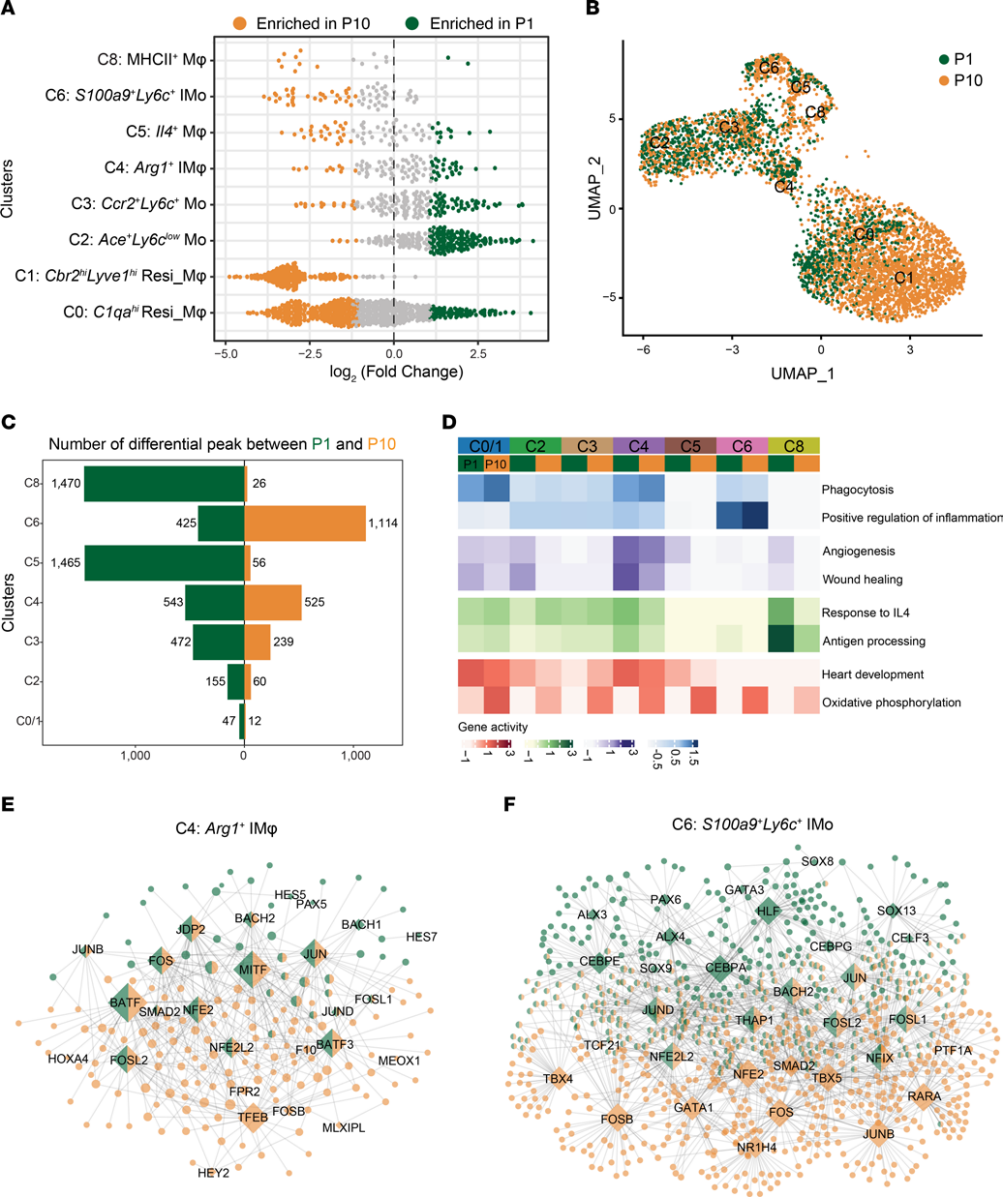
Comparative analysis of epigenetic features in MPC subclusters between P1 and P10. (**A**) Bee swarm plot showing the distribution of log fold change in abundance between P1 and P10 hearts across different clusters. Differential abundance neighborhoods at FDR 50% are colored. (**B**) UMAP embedding of H3K27ac CoBATCH dataset colored by stages in Figure 1B. (**C**) Number of differential H3K27ac ChIP-Seq peaks between P1 (green) and P10 (orange) hearts in each subcluster. (**D**) Heatmap displaying average gene activities of nearby genes participating in typical functions among all clusters from P1 and P10 hearts. (**E** and **F**) TF regulatory network showing specific and shared key TFs and their target genes (TGs) between P1 (green) and P10 (orange) hearts in C4 (**E**) and C6 (**F**). The edges indicate TF-TG pairs, and the size of the dot indicates the number of nodes in the network. The green and orange bicolor represents TFs and TGs shared by P1 or P10 cells.

To explore the regulatory mechanisms governing heart regenerative potential, comprehensive comparative analyses between P1 and P10 cells were conducted. Differential abundance analysis of cells from P1 and P10 hearts in each cluster using Milo (49) revealed that *Cbr2*^hi^*Lyve1*^hi^ C1 and *S100a9*^+^*Ly6c*^+^ C6 cells were exclusively enriched in P10 hearts (Figure 5A). Consistent with previous immunostaining results (Figure 2, B and C), we observed the *Arg1*^+^ C4 population was more abundant in P1 than in P10 hearts (Figure 5A). Additionally, the antiinflammatory *Ly6c*^lo^ nonclassical monocytes (C2 cluster) were also enriched in P1 hearts (Figure 5A).

When projecting the sample information onto the single-cell UMAP, we observed distinct distributions of cells from P1 and P10 hearts even within the same cluster (Figure 5B). This observation prompted us to explore the epigenetic heterogeneities within each subcluster. Comparative analysis of the H3K27ac signals between P1 and P10 cells within each cluster identified variable numbers of peaks between the 2 stages, with *S100a9*^+^*Ly6c*^+^ C6 showing the largest number of P10-enriched peaks (Figure 5C). To further explore the biological significance of differential enhancer peaks within subclusters, we examined the enrichment of enhancer scores for marker genes involved in typical functions. Interestingly, P10 cells in the *S100a9*^+^*Ly6c*^+^ C6 cluster exhibited higher proinflammatory activities, while P1 cells in *Arg1*^+^ C4 were more enriched in processes related to angiogenesis and wound healing (Figure 5D). Taken together, these findings reveal distinct injury-induced responses in P1 and P10 hearts, even within the same subpopulation. Additionally, the prorepair potential of P1 cells and the proinflammatory potential of P10 cells, as reflected by H3K27ac signals, may be responsible for the distinct regeneration abilities.

Inspired by the epigenetic heterogeneities between P1 and P10 cells, we next explored their differences in TF-TG networks across all clusters (Supplemental Table 10). Overall, the shared TF-TG pairs were more frequently detected in *Arg1*^+^ C4 cluster, suggesting relatively less variance in TF-TG usage between P1 and P10 cells in this cluster (Figure 5, E and F and Supplemental Figure 6). In contrast, P1 and P10 cells exhibited a greater number of unique TF-TG regulatory networks in C2 and C3 clusters (Supplemental Figure 6, C and D). Additionally, we observed the TF-TG networks centered on CEBP transcription factors, crucial for macrophage development (50), were enriched in P1 cells, whereas FOS and JUN inflammatory transcription factor–centered TF-TG networks were frequently detected in P10 cells within the *S100a9*^+^*Ly6c*^+^ C6 cluster (Figure 5F).

Collectively, the specific generation of the inflammatory *S100a9*^+^*Ly6c*^+^ C6 cluster in P10 hearts after MI, along with the differential epigenomic H3K27ac chromatin states responding to MI in regenerative P1 and nonregenerative P10 hearts within each subpopulation, underscores the disparity in reparative capacity. These data also emphasize the unique TF-TG regulatory networks in regenerative hearts within each cluster.

### Functional evaluation of S100a9^+^Ly6c^+^ IMos and Arg1^+^ IMφs on neonatal heart repair after MI.

**Figure 6:**
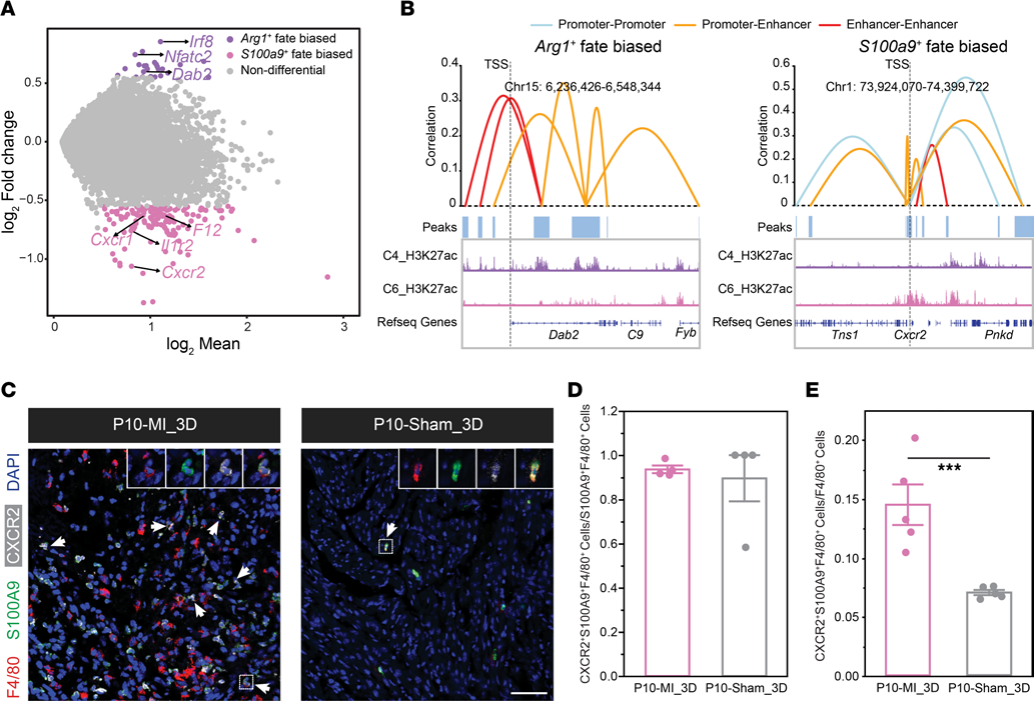
Differential analysis of gene programs orchestrating the generation of *Arg1*^+^ C4 and *S100a9*^+^*Ly6c*^+^ C6 cells. (**A**) MA plot comparing H3K27ac signals between MI-induced *Arg1^+^* IMφs and *S100a9^+^ Ly6c*^+^ IMos. H3K27ac signals were counted by read density 50 kb upstream and 30 kb downstream of gene body and normalized by read depth. (**B**) CCRNs between H3K27ac peaks near *Dab2* and *Cxcr2* loci in the *Arg1^+^* and *S100a9^+^* biased fates, respectively. Peak regions were indicated as blue boxes. Grey dotted lines indicate TSS of *Dab2* and *Cxcr2,* respectively. (**C**) Immunostaining for F4/80, S100A9, and CXCR2 in the IZ of P10-MI/sham_3D hearts to validate the expression of CXCR2 in *S100a9*^+^*Ly6c*^+^ IMos. Scale bar: 50 μM. (**D**) Quantification of the percentage of CXCR2^+^S100A9^+^F4/80^+^ cells in S100A9^+^F4/80^+^ cells in **C**. (**E**) Quantification of the percentage of CXCR2^+^S100A9^+^F4/80^+^ cells in F4/80^+^ cells in **C**. The *P* value was determined by paired 2-tailed Student’s *t* test (**D** and **E**). Data represent mean ± SEM. ***P < 0.001.

Given the significant induction of *Arg1*^+^ IMφ C4 and *S100a9*^+^ IMo C6 cells following injury, we were intrigued by the regulatory mechanisms governing the monocyte fate specification toward C4 or C6. By comparing the H3K27ac signals, we identified genes displaying *Arg1*- or *S100a9-*biased activation (Supplemental Table 11). For example, *Dab2* has been demonstrated to participate in promoting tissue repair and reducing inflammation (51). Consistently, the enhancer activities of *Dab2* were highly enriched in the *Arg1*-biased fate. In contrast, the proinflammatory chemokine CXCR2 gene locus exhibited significant enrichment of enhancer activities toward the *S100a9*-biased fate (52) (Figure 6A). The differential enhancer activities of *Dab2* and *Cxcr2* in the 2 subclusters were further corroborated by the distinct CCRNs of H3K27ac peaks surrounding individual gene loci (Figure 6B). Further, the expression of CXCR2 in *S100a9*^+^*Ly6c*^+^ IMos was evident in the P10-MI_3D hearts, and the generation of CXCR2^+^S100A9^+^F4/80^+^ cells was notably induced in the IZ of P10-MI_3D hearts compared with sham hearts (Figure 6, C–E). In summary, the generation of monocyte-related *Arg1*^+^ C4 and *S100a9*^+^*Ly6c*^+^ C6 clusters after MI was orchestrated by 2 distinct sets of gene programs.

**Figure 7:**
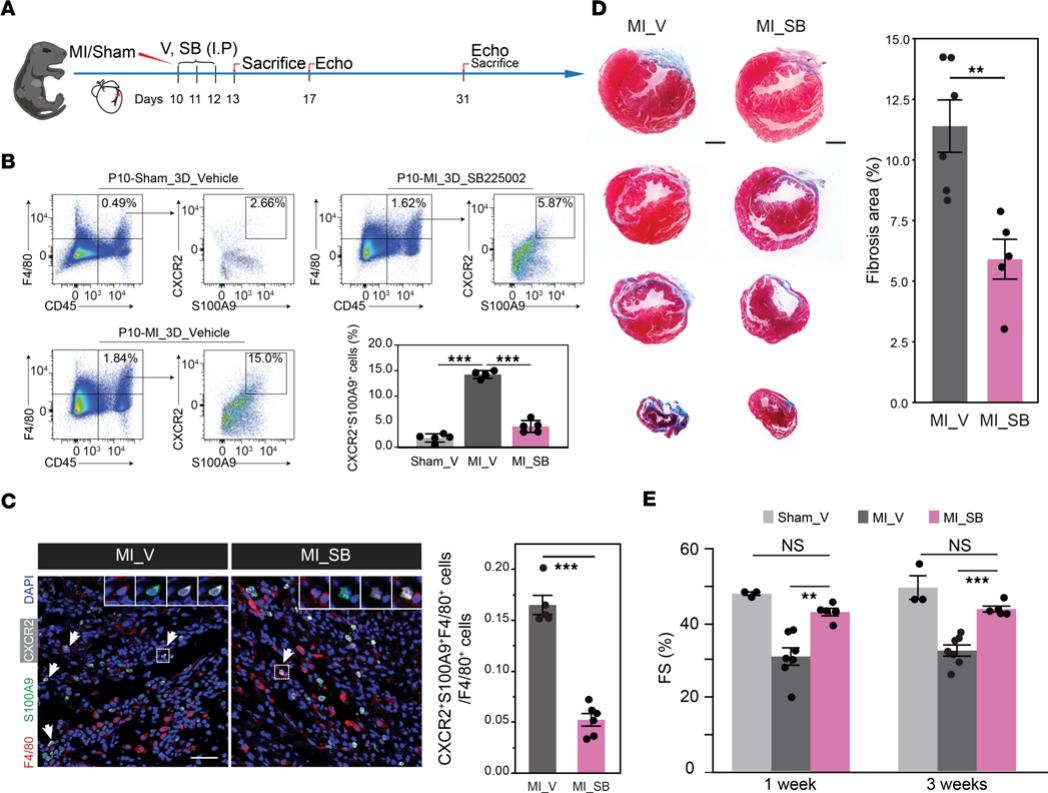
Targeting *S100a9*^+^*Ly6c*^+^ C6 cells by inhibition of CXCR2 improves heart function after MI. (**A**) Schematic representation of targeting *S100a9*^+^*Ly6c*^+^ IMos experimental design. SB225002 (SB) or vehicle (V) was i.p. injected for 3 days immediately after P10 MI. (**B**) Flow cytometry showing the percentage of S100A9^+^CXCR2^+^ IMos in each treatment group in **A**. (**C**) Representative immunostaining and quantification of S100A9^+^CXCR2^+^F4/80^+^ cells in the IZ of P10-MI_3D hearts injected with SB225002 (SB) or vehicle (V). Scale bar: 50 μM. (**D**) Masson trichrome staining of cross sections from hearts injected with SB225002 (SB) or vehicle (V) and quantification analysis. Scale bar: 200 μM. (**E**) Echocardiographic measurements of heart function at 1- and 3 weeks post P10 MI. The *P* value was determined by 1-way ANOVA with post hoc Dunnett’s test (**B** and **E**), and unpaired 2-tailed Student’s *t* test (**C** and **D**). Data represent mean ± SEM. *P < 0.05; **P < 0.01; ***P < 0.001.

Considering that the generation of the proinflammatory *S100a9*^+^*Ly6c^+^* IMo subset was significantly induced after MI in P10 but not in P1 hearts, we sought to determine whether targeting this subpopulation could potentially enhance cardiac function after MI induced at P10. To test this hypothesis, we opted to selectively target CXCR2 chemokines in the infarcted hearts, as CXCR2 was highly enriched in the *S100a9-*biased fate (Figure 6). Therefore, we induced MI at P10 and subjected the mice to a 3-day treatment with 5 mg/kg SB225002, a CXCR2 receptor antagonist, to block the CXCR2 signaling pathway in the *S100a9*^+^*Ly6c^+^* IMo subpopulation (Figure 7A). Flow cytometry and immunostaining for F4/80, S100A9, and CXCR2 in P10-MI_3D injured hearts confirmed a significant reduction in *S100a9*^+^*Ly6c^+^* IMos infiltration after CXCR2 inhibition (Figure 7, B and C). As a result, dramatically reduced fibrosis and improved cardiac function could be observed in injured hearts after CXCR2 blockade compared with the vehicle groups (Figure 7, D and E).

**Figure 8:**
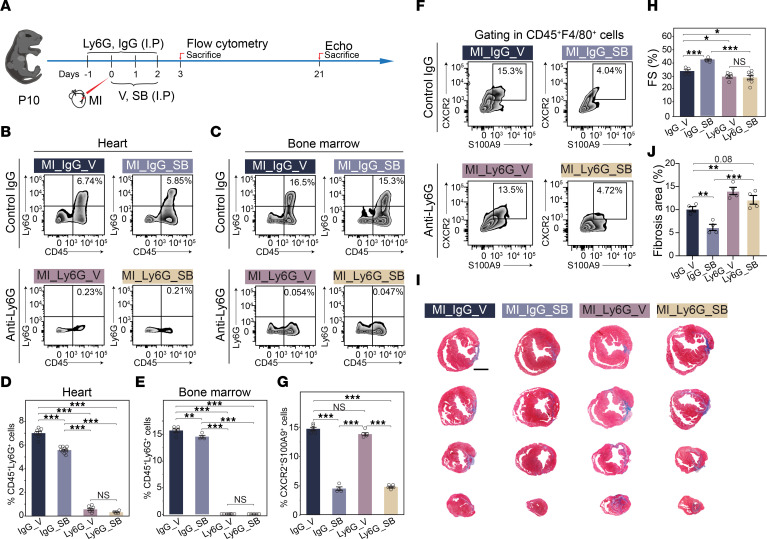
The beneficial effect of CXCR2 inhibition on cardiac repair is nullified by neutrophil depletion. (**A**) Schematic representation of the experimental design targeting *S100a9*^+^*Ly6c*^+^ IMos in mice treated with anti-Ly6G antibody or IgG isotype. (**B** and **C**) Flow cytometry plots of CD45^+^Ly6G^+^ cells in the heart (**B**) and bone marrow (**C**) of mice treated with SB225002/vehicle and anti-Ly6G/IgG antibodies as shown in **A**. (**D** and **E**) Quantification analysis of flow cytometry results in **B** and **C**. (**F** and **G**) Flow cytometry plots (**F**) and quantification analysis (**G**) of the percentage of S100A9^+^CXCR2^+^ IMos in **A**. (**H**) Echocardiographic measurements of heart function 3 weeks after P10 MI treated with SB225002/vehicle and anti-Ly6G/IgG antibodies as shown in **A**. (**I** and **J**) Representative Masson trichrome staining (**I**) of cross-sections from hearts described in **A** and quantification analysis (**J**). Scale bar: 200 μM. The *P* value was determined by 1-way ANOVA with post hoc Scheffe’s test (**D**, **E** and **G**), or with post hoc LSD test (**H** and **J**). *P < 0.05; **P < 0.01; ***P < 0.001.

Since CXCR2 participates in regulating neutrophil recruitment (53), we further examined the effect of CXCR2 inhibition on cardiac repair in mice lacking neutrophils (Figure 8A). P10 mice were treated with anti-Ly6G monoclonal antibodies on days –1, 0, 1, 2, and 3 after MI, resulting in efficient neutrophil depletion in both the heart and bone marrow (54, 55) (Figure 8, B–E). As a result, we found that the abundance of the *S100a9*^+^*Ly6c^+^* C6 population remained unaffected after neutrophil depletion (Figure 8, F and G), confirming its monocyte rather than neutrophil identity. Additionally, neutrophil depletion by anti-Ly6G antibody worsened cardiac function and increased cardiac fibrosis 3 weeks after P10 MI (Figure 8, H–J), consistent with observations in adult neutrophil-depleted mice following MI (56). Unexpectedly, we observed that the enhanced cardiac repair capacity in P10 hearts from CXCR2 inhibition was blocked by neutrophil depletion (Figure 8, H–J), indicating that the positive effects of *S100a9*^+^*Ly6c*^+^ monocyte depletion on cardiac repair are possibly overshadowed by neutrophil depletion.

To obtain direct evidence of the function of *S100a9*^+^*Ly6c^+^* IMos in neonatal heart repair, we injected FACS-sorted *S100a9*^+^*Ly6c^+^* IMos, along with FACS-sorted *Lyve1*^+^F4/80^+^ cardiac-resident macrophages (C0 and C1) as a control, into the myocardium of P5 mice immediately after ligation of the left anterior descending coronary artery (Supplemental Figure 7A). Reintroduction of *S100a9*^+^*Ly6c^+^* IMos, but not *Lyve1*^+^F4/80^+^ macrophages, resulted in a notable increase in cardiac infarct size and a reduction in cardiac function compared with the PBS group (Supplemental Figure 7, B–E). This was accompanied by a significantly increased expression of proinflammatory cytokines, such as TNF-α, S100A8/9, IL-6 and IL-1B in *S100a9*^+^*Ly6c^+^* C6–injected hearts. However, the expression TNFR1, TNF-α, and IL-1B exhibited an opposite pattern in *Lyve1*^+^F4/80^+^ macrophage-injected hearts (Supplemental Figure 7F). This observation solidifies the detrimental role of *S100a9*^+^*Ly6c^+^* C6 cells in the cardiac repair process after MI, further supporting the possibility that the beneficial effect of CXCR2 blockade on cardiac repair is more likely due to the decreased generation of *S100a9*^+^*Ly6c*^+^ C6 cells rather than reduced neutrophil recruitment. However, inhibition of CXCR2 did not impact P1 heart repair after MI (Supplemental Figure 8, A–E), as evidenced by comparable heart function and fibrotic states between hearts with CXCR2 inhibition and control hearts 1 month after P1 injury (Supplemental Figure 8, C–E). The distinct impacts of CXCR2 inhibition on P1 and P10 hearts may stem from the relatively lower abundance of *S100a9*^+^*Ly6c*^+^ C6 cells in P1 hearts after injury (Figure 2, D and E and Supplemental Figure 4A).

As the prorepair *Arg1*^+^ C4 cluster was more enriched in P1 hearts than in P10 hearts (Figure 2, B and C and Supplemental Figure 4, H and I), we proceeded to examine its role in P1 heart repair after injury. A 6-day treatment of the P1 mice with nor-NOHA monoacetate, a selective ARG1 inhibitor, significantly reduced the generation of *Arg1*^+^ C4 cells after MI (Supplemental Figure 8, F and G). Consequently, cardiac function, assessed by fractional shortening a month after P1 injury, was significantly reduced in mice with ARG1 inhibition (Supplemental Figure 8H). Further, trichrome staining at the same time point revealed increased fibrotic cardiac areas in mice that received nor-NOHA monoacetate treatment (Supplemental Figure 8, I and J). Taken together, these data suggest that *S100a9*^+^*Ly6c^+^* C6 cells, which are specifically generated in nonregenerative P10 heart after injury, impede neonatal heart repair after injury. Blocking these cells in P10 injured hearts significantly improved heart repair capacity. Moreover, the prorepair *Arg1*^+^ C4 cells are essential for the spontaneous reparative capacity of P1 hearts.

### S100a9^+^Ly6c^+^ IMos suppression promotes proliferation and protective activities in cardiomyocytes.

**Figure 9:**
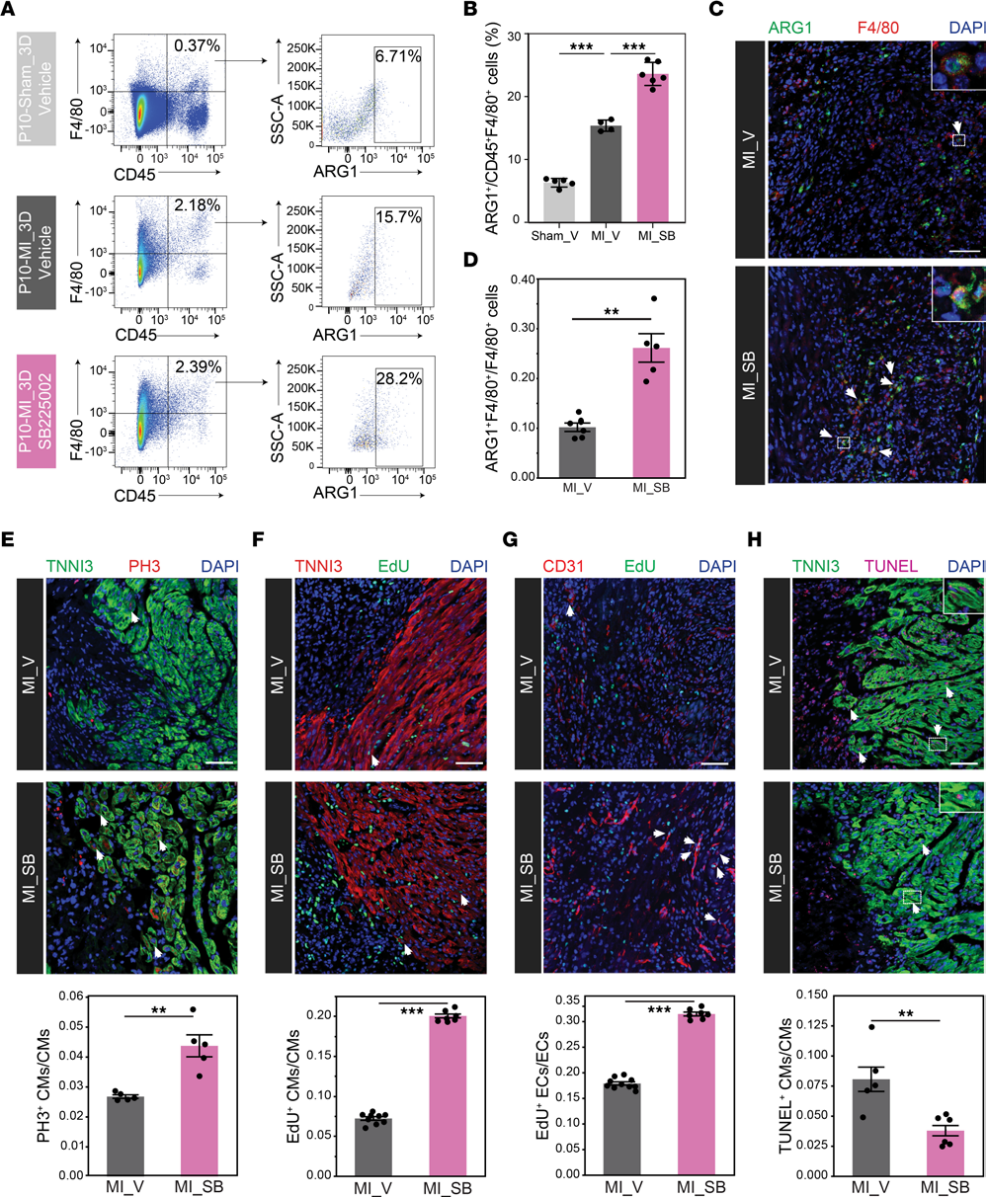
Characterization of the cellular mechanisms underlying improved heart repair capacity after MI following targeting of *S100a9*^+^*Ly6c*^+^ IMos. (**A** and **B**) Flow cytometry plot (**A**) and quantification analysis (**B**) of the percentage of ARG1^+^F4/80^+^ IMφ in P10-MI_3D hearts injected with SB225002 (SB) or vehicle (V) and P10-sham_3D hearts injected with vehicle (V). (**C** and **D**) Representative immunostaining (**C**) and quantification (**D**) for ARG1^+^F4/80^+^ cells in the IZ of P10-MI_3D hearts injected with SB225002 (SB) or vehicle (V). (**E** and **F**) Representative immunostaining and quantification for TNNI3^+^PH3^+^ (**E**) and TNNI3^+^EdU^+^ (**F**) proliferative CMs in the BZ of P10-MI_3D hearts injected with SB225002 (SB) or vehicle (V). (**G**) Representative immunostaining and quantification for CD31^+^EdU^+^ proliferative ECs in the BZ of P10-MI_3D hearts injected with SB225002 (SB) or vehicle (V). (**H**) Representative images of TUNEL assay for the BZ of P10-MI_3D hearts injected with SB225002 (SB) or vehicle (V). *n* = 4–9 mice per experimental group. Scale bars: 50 μM (**C**, **E**, **F**, **G** and **H**). The *P* value was determined by 1-way ANOVA with post hoc Dunnett’s test (**B**), or paired (**E**) and unpaired (**D**, **F**, **G** and **H**) 2-tailed Student’s *t* test. Data represent mean ± SEM. *P < 0.05; **P < 0.01; ***P < 0.001.

To explore the cellular mechanism underlying the enhanced reparative capacity of P10 hearts following CXCR2 inhibition, we first examined the number of *Arg1*^+^ macrophages based on the hypothesis that the inhibition of the generation of *S100a9*^+^*Ly6c^+^* IMo would increase the lineage specification toward *Arg1*^+^ C4 from monocytes. Indeed, we observed an approximately 2.5-fold increase in the number of ARG1^+^ macrophages in the IZ of SB225002-treated hearts (Figure 9, A–D). This increase potentially contributes to an enhanced capacity for cardiac repair, as *Arg1*^+^-infiltrated Mφs exhibited the strongest wound healing and angiogenesis-related functions. Importantly, elevated cardiomyocyte (CM) proliferation in the BZ of P10-MI_3D hearts following SB225002 treatment became evident through immunostaining of EdU, PH3, and KI67 (Figure 9, E and F and Supplemental Figure 9A). Moreover, an increase in the number of CD31^+^ endothelial cells was observed in the BZ after CXCR2 inhibition (Figure 9G and Supplemental Figure 9, B and C). This effect could potentially be attributed to the elevated production of IGF1 by *Arg1*^+^-infiltrated Mφs after CXCR2 inhibition (57) (Supplemental Figure 2E). To study the effects of CXCR2 blockade on apoptosis, we determined the frequency of TUNEL^+^ CMs. The frequency of TUNEL^+^ CMs was 2.5-fold lower in the SB225002 treatment group than in the vehicle group (Figure 9H), suggesting increased CM survival coincident with the decreased inflammatory response in infarcted hearts after blocking *S100a9*^+^*Ly6c^+^* IMos (58). Taken together, these data suggest that targeting the proinflammatory *S100a9*^+^*Ly6c^+^* IMos with a CXCR2 inhibitor resulted in significant improvements in myocardial outcomes through the increased proliferation of CMs and ECs, along with increased survival of CMs during cardiac repair.

### scRNA-Seq reveals molecular mechanisms underlying the improved cardiac function in infarcted hearts after CXCR2 blockade.

**Figure 10:**
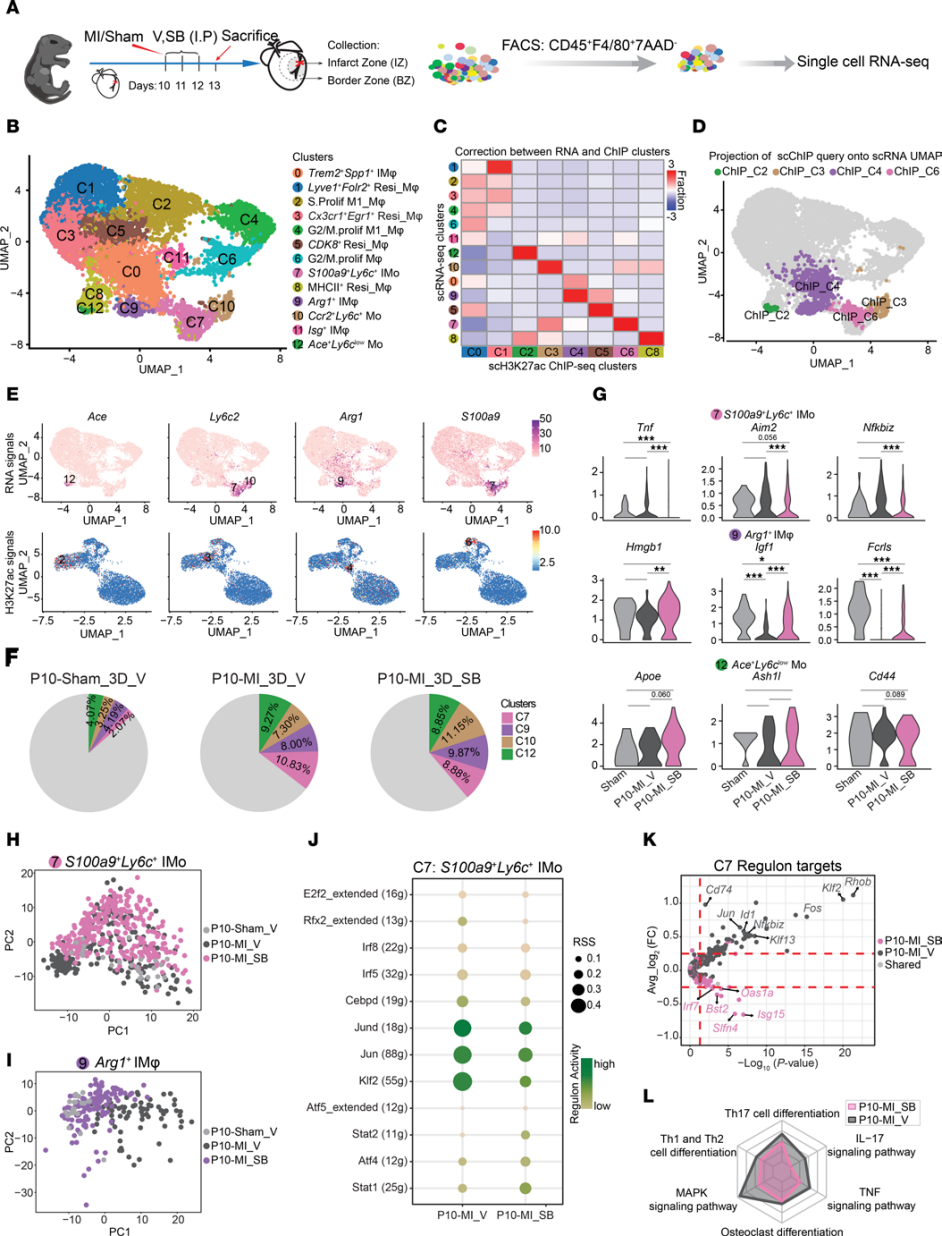
Single-cell RNA-Seq of macrophages/monocytes reveals the molecular basis underlying the protective functions of CXCR2 blockade after MI. (**A**) Schematic representation of the experimental design. The IZ and BZ of hearts were collected after daily injection of SB225002 (SB) or vehicle (V) for 3 days after P10 MI. (**B**) UMAP plot of 8,571 cardiac CD45^+^F4/80^+^ mononuclear phagocytic cells. (**C**) Heatmap displaying the fraction of cells in each scRNA-Seq cluster linked to corresponding ChIP-Seq clusters through integration by Seurat V3. The color bar represents values normalized by *z* score for each column. (**D**) UMAP showing single-cell H3K27ac ChIP identified monocyte-related clusters embedding onto the scRNA-Seq UMAP. (**E**) Feature plots showing the representative scRNA signals (top) and scH3K27ac signals (bottom) of monocyte-related marker genes. (**F**) Pie charts showing the percentage of scRNA clusters C7, C9, C10, and C12 in each experimental condition. (**G**) Violin plots showing the RNA signals of representative genes related to typical functions in C7, C9, and C12 under 3 different experimental conditions. The *P* value was calculated by Kruskal-Wallis *H* test, followed by Dunn’s test. *P < 0.05; **P < 0.01; ***P < 0.001. (**H** and **I**) PCA showing the distribution of cells from P10-sham_V, P10-MI_SB and P10-MI_V hearts in C7 (**H**) and C9 (**I**) clusters. (**J**) Dotplot displaying the scaled activity scores of regulons for cells from P10-MI_V and P10-MI_SB hearts in C7. The dot size indicates the regulon specificity score (RSS) and the color indicates the Z-score of the regulon activities. (**K**) Volcano plot showing the differentially expressed regulon’s target genes in **J** between P10-MI_V and P10-MI_SB hearts. The red dashed line represents the threshold of differential expression, |log2FC| > 0.25 and *P* value < 0.05. (**L**) Radar chart displaying the enrichment of regulon’s target genes in **K** in inflammatory-related pathways between P10-MI_V and P10-MI_SB hearts.

To identify the molecular mechanisms underlying the improvement in cardiac function resulting from targeting *S100a9*^+^*Ly6c^+^* IMos, we performed scRNA-Seq on CD45^+^F4/80^+^ MPCs isolated from the IZ and BZ of P10-sham_vehicle, P10-MI_vehicle, and P10-MI_SB225002 hearts at 3 days after P10-MI (Figure 10A). A total of 10,546 cells passed quality control and were divided into 17 clusters (Supplemental Figure 10A and Supplemental Table 12). Based on the expression of MPC marker genes (*Adgre1, Cx3cr1, Ly6c2, Ccr2*) and non-MPC marker genes (*Kdr*, *Ms4a1*, *Col1a1*, *Lef1*, *S100a9*) (Supplemental Figure 10, B and C), clusters 0, 1, 2, 3, 4, 5, 6, 10, 11, 13, and 15 were defined as MPCs and selected for further analysis, comprising a total of 8,571 cells (P10-sham_vehicle: 2,045 cells; P10-MI_vehicle: 2,764 cells; P10-MI_ SB225002: 3,762 cells). By reclustering the MPCs, we obtained 13 subpopulations and annotated their identity according to the specific gene expression patterns observed within each cluster (Figure 10B, Supplemental Figure 10, D and E, and Supplemental Table 13).

To establish a connection between the ChIP-Seq annotated monocyte-related clusters (C2, C3, C4 and C6) and the scRNA-Seq clusters, we performed integrated analysis of the scChIP-Seq and scRNA-Seq datasets using canonical correlation analysis (CCA) by Seurat V3 (19). The annotations from the 2 datasets were highly consistent for monocyte-related clusters (*Ace*^+^*Ly6c*^lo^ Mo, *Ccr2*^+^*Ly6c*^+^ Mo, *Arg1*^+^ IMφ, and *S100a9*^+^*Ly6c*^+^ IMo) (Figure 10, C and D and Supplemental Table 14). This alignment further confirmed the enhancer activities and the expression levels of cluster-specific marker genes (Figure 10E). Consistent with previous studies (10, 12), our scRNA-Seq dataset also revealed a substantial increase in monocyte-related cells (RNA: C7, C9, C10, and C12) after MI in P10 hearts (Figure 10F and Supplemental Figure 10E). Notably, the proportion of cluster 7 (*S100a9*^+^*Ly6c*^+^ IMo) decreased, while that of cluster 9 (*Arg1*^+^ IMφ) increased after SB225002 treatment compared with vehicle treatment, consistent with our immunostaining and flow cytometry results (Figure 9, A–D and Supplemental Figure 11A). Prompted by the observed heterogeneities within each subcluster (Figure 2A), we next asked whether there were differences in gene expression patterns between the CXCR2 inhibition and control groups within the same subcluster. Among the 3 monocyte-derived clusters, CXCR2 blockade led to a decrease in proinflammatory-related gene expression within the C7 cluster, while it increased the expression of wound repair–related genes in the C9 cluster (Figure 10G). Overall, we observed that P10-MI_ SB225002 cells displayed a closer resemblance to cells from the sham group than to P10-MI_vehicle cells in both C7 and C9 clusters (Figure 10, H and I).

We further applied single-cell regulatory network inference and clustering (SCENIC) to explore the regulons differentially enriched in P10-MI_SB225002 relative to control groups in both C7 and C9 clusters (59). As a result, decreased enrichment for inflammatory-related regulons in P10 hearts after SB225002 treatment was observed in C7 cluster (Figure 10J and Supplemental Table 15). Moreover, 7 upregulated and 61 downregulated target genes of C7 regulons in P10-MI_SB225002 compared with control hearts was identified (Figure 10K and Supplemental Table 16), and these targets exhibited reduced enrichment in signaling pathways involved in inflammatory responses after CXCR2 blockade (Figure 10L and Supplemental Table 17). However, we did not identify differential regulons directly involved in wound healing or angiogenesis processes between P10-MI_SB225002 and control hearts in the C9 cluster (Supplemental Figure 11B and Supplemental Table 15). In summary, these findings supported the observations that targeting *S100a9*^+^*Ly6c^+^* IMos enhances the reparative capacity after MI in P10 hearts and elucidated the underlying molecular mechanisms.

In addition, differential gene expression analysis of aggregated pseudobulk MPCs from SB225002- and vehicle-treated P10-MI hearts demonstrated a significant reduction in the expression of proinflammatory genes (*Fos* and *Jun*) and an increase in the expression of reparative genes (*Fn1* and *Trem2*) following CXCR2 inhibition (Supplemental Figure 12, A and B and Supplemental Table 18). Gene ontology analysis of the differentially expressed genes further supported these observations (Supplemental Figure 12C and Supplemental Table 19). Consistent with the decreased inflammatory responses in SB225002-treated hearts, KEGG analysis revealed the deactivation of multiple inflammatory signaling pathways, including the IL-17, MAPK, and TNF pathways, following CXCR2 blockade (Supplemental Figure 12D). In summary, our scRNA-Seq analysis demonstrated that the reduction in the proportion of the proinflammatory subpopulation and the increase in the proportion of the reparative subpopulation, coupled with the differential expression of the corresponding functional genes, collectively contributed to the enhanced reparative potential in hearts after P10 MI hearts upon targeting *S100a9*^+^*Ly6c^+^* IMos.

### Single-cell H3K27ac ChIP-Seq reveals the myeloid composition in the adult heart after MI.

**Figure 11:**
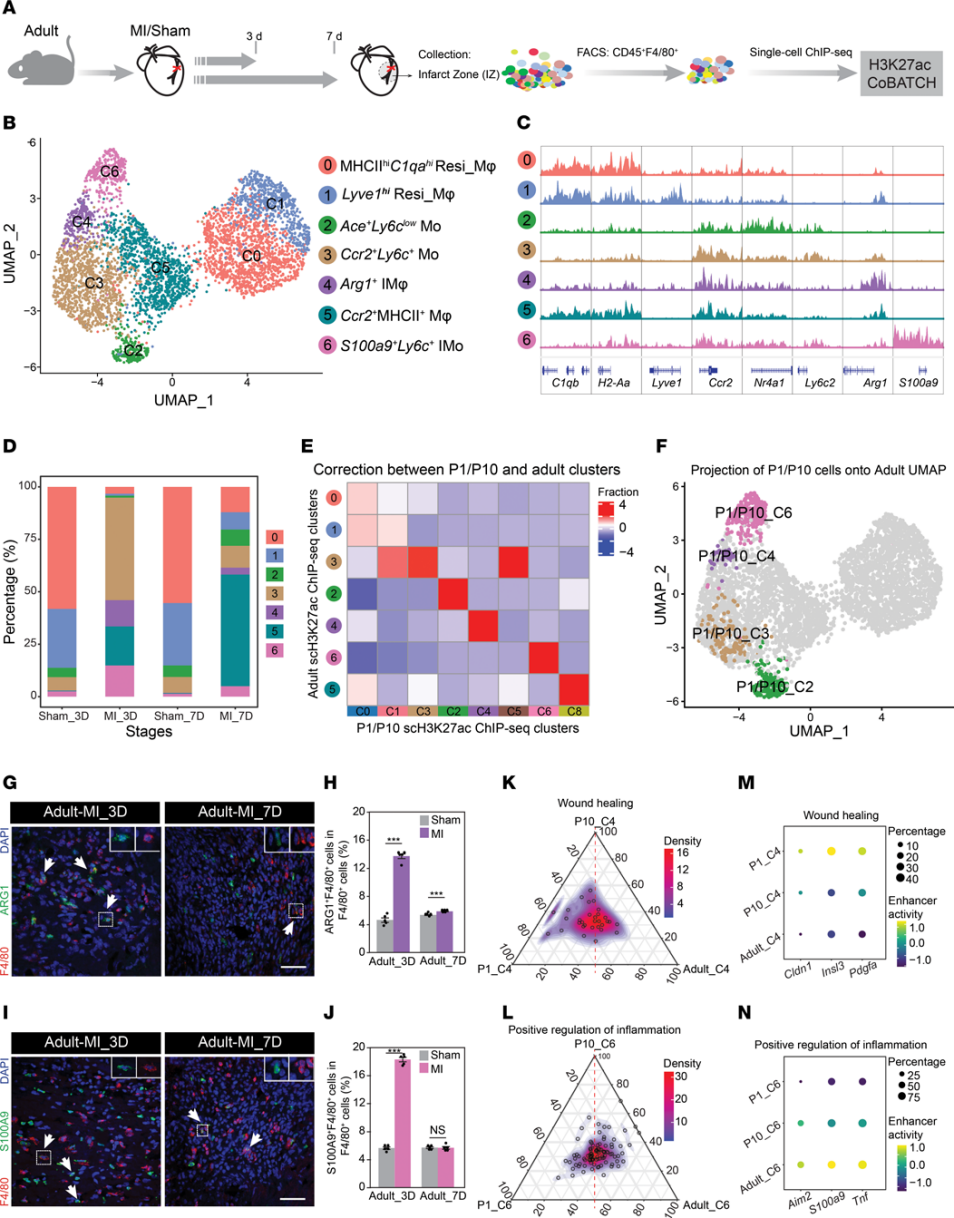
Single-cell H3K27ac ChIP-Seq of macrophages/monocytes in adult hearts at 3 and 7 days after MI/sham. (**A**) Schematic representation of the experimental design. (**B**) UMAP plot of 4,613 cardiac CD45^+^F4/80^+^ mononuclear phagocytic cells from adult hearts 3 and 7 days after MI/sham identified 7 clusters. (**C**) Genome browser view of H3K27ac signals around cluster-specific marker genes. (**D**) Bar plot showing proportions of each cluster according to experimental conditions. (**E**) Heatmap displaying the fraction of cells in each adult cluster linked to corresponding P1/P10 H3K27ac ChIP-identified clusters through integration by Seurat V3. The color bar represents values normalized by *z* score for each column. (**F**) UMAP showing P1/P10 ChIP-identified monocyte-related clusters embedding onto the adult scChIP-Seq UMAP. (**G** and **H**) Representative immunostaining (**G**) and quantification (**H**) of ARG1^+^F4/80^+^ cells in the IZ of Adult-MI_3D hearts. (**I** and **J**) Representative immunostaining (**I**) and quantification (**J**) of S100A9^+^F4/80^+^ cells in the IZ of Adult-MI_3D hearts. (**K** and **L**) Ternary plot showing stage-specific enhancer activities of genes involved in wound healing (**K**) and proinflammatory activities (**L**) in C4 and C6, respectively. The red dashed line represents the central axis between P1 and adult stages and the circles represent genes. (**M** and **N**) Dot plots displaying the enhancer activities of representative genes related to wound healing functions in *Arg1^+^* IMφ (**M**) and proinflammatory functions in *S100a9*^+^*Ly6c^+^* IMo (**N**) among 3 stages. Scale bar: 50 μM. *n* = 5 mice per experimental group. The *P* value was determined by unpaired 2-tailed Student’s *t* test. Data represent mean ± SEM. ***P < 0.001.

To examine the existence of *Arg1*^+^ C4 and *S100a9*^+^*Ly6c*^+^ C6 cells in adult mouse hearts after MI, we further conducted H3K27ac CoBATCH on CD45^+^F4/80^+^7AAD^–^ MPCs isolated 3 and 7 days after MI from the IZ of adult mouse hearts (Figure 11A). After quality control filtering, 4,613 cells, with 1,864 from sham and 2,749 from MI, were subjected to Seurat clustering, resulting in 7 epigenetically distinct populations (Figure 11B and Supplemental Table 20). Based on the enhancer signal distributions of marker genes, we annotated them as follows: MHCII^hi^*C1qa^hi^* Resi_Mφ (cluster 0), *Lyve1^hi^* resident cardiac macrophages (cluster 1), *Ace^+^Ly6c^lo^* monocytes (cluster 2), *Ccr2^+^Ly6c^+^* monocytes (cluster 3), *Arg1^+^* infiltrating macrophages (cluster 4), *Ccr2^+^*MHCII^+^ macrophages (cluster 5), and *S100a9^+^Ly6c^+^* infiltrating monocytes (cluster 6) (Figure 11, B and C and Supplemental Figure 13A). Interestingly, when comparing this adult dataset with the P1/P10 dataset, we were unable to identify the *Il4*^+^ macrophages but detected the existence of *Ccr2*^+^MHCII^+^ macrophages specifically generated after MI (Figure 1B, Figure 11, B–D, and Supplemental Figure 13, B and C), consistent with studies reported by others (9, 13). This suggests that *Il4*^+^ macrophages specifically exist in neonatal mouse hearts, while *Ccr2*^+^MHCII^+^ macrophages are uniquely generated in adult hearts after MI.

Consistent with observations in P10 hearts, we found that the generation of *Arg1*^+^ C4 cells and *S100a9*^+^*Ly6c*^+^ C6 cells was significantly induced following adult MI (Figure 11D and Supplemental Figure 13, B and C). Moreover, integrated analysis of the P1/P10 and adult datasets using CCA by Seurat V3 verified that resemblance of both *Arg1*^+^ macrophages and *S100a9*^+^*Ly6c*^+^ monocytes defined by the 2 datasets (Figure 11, E and F), confirming the similar phenomena observed in P10 hearts (Figure 2, B–E). Additionally, we performed immunofluorescence staining to confirm the induction of *Arg1*^+^ and *S100a9*^+^*Ly6c*^+^ populations in adult mouse hearts 3 days after MI (Figure11, G–J). Examination of the enhancer activities of genes participating in wound healing function among *Arg1*^+^ cells from different stages reveals the highest enrichment in P1 hearts (Figure 11K), while the *S100a9*^+^*Ly6c*^+^ cells from adult hearts exhibited the strongest enhancer activities adjacent to genes involved in proinflammatory activities (Figure 11L), as validated by the comparison of enhancer activities alongside representative marker genes (Figure 11, M and N).

Altogether, analysis of the H3K27ac CoBATCH data revealed that the overall myeloid composition in the adult heart after MI resembled that observed in the neonatal P10 hearts, except for the *Il4*^+^ and *Ccr2*^+^MHCII^+^ macrophages. However, *S100a9*^+^*Ly6c*^+^ cells from adult hearts exhibit the strongest proinflammatory activities, perhaps contributing to their poor regenerative capacity.

## Discussion

In summary, we successfully generated the first spatiotemporal single-cell resolution epigenomic map of macrophages in regenerative (P1) and nonregenerative (P10) mouse hearts following MI. Utilizing this comprehensive dataset, we identified enhancer regulatory networks specific to individual cell types, examined the enrichment of SNPs linked to cardiovascular diseases, delineated the regulatory trajectories, and identified essential transcription factors governing the fate and functional specifications of each cluster. Remarkably, our study revealed the existence of epigenetic heterogeneity within each subcluster, which partially accounts for the distinct regeneration potential of P1 and P10 hearts (Figure 2A and Figure 5). Significantly, we identified a granulocyte-like proinflammatory population of *S100a9*^+^*Ly6c*^+^–infiltrated monocytes that were specifically enriched in P10 and adult hearts after MI. SB225002-mediated therapeutic inhibition of CXCR2, to target the *S100a9*^+^*Ly6c*^+^ subpopulation, showed significant improvements in cardiac function and notable reductions in fibrosis after MI. Additional scRNA-Seq analysis of hearts treated with SB225002 and vehicle controls corroborated the cellular and molecular mechanisms underlying the elevated cardiac function resulting from CXCR2 inhibition. Our findings provide a valuable resource to explore the molecular mechanisms underlying the function and fate specification of distinct MPC subtypes for cardiac repair after MI, which has the potential to guide the development of novel therapeutic strategies aimed at targeting heart failure after MI.

Recent advancements in scRNA-Seq have greatly facilitated the exploration of macrophage heterogeneity in adult mouse hearts after MI (10–14). However, the comprehension of epigenetic heterogeneity and the molecular foundation that underlies the diversity of macrophage subtypes remain rather limited. Chromatin states, such as enhancer elements, encode and specify cell fates (60), offering a promising avenue for tracing cell differentiation paths, whether discrete or along a continuum. In light of this, we undertook an analysis of the epigenetic heterogeneity of cardiac macrophages through single-cell ChIP-Seq for H3K27ac, a marker of active enhancers (18). Unexpectedly, we did not discern prominent enhancer signals surrounding *Timd4*, a marker of tissue-resident macrophages identified by scRNA-Seq (13), within our resident C0 and C1 clusters. This discrepancy could stem from the possibility that *Timd4* expression is not controlled by H3K27ac signals but by other epigenetic mechanisms. Further, we failed to identify *Ccr2*^hi^MHCII^hi^ macrophages in the neonatal P1 and P10 hearts, but detected them in the adult hearts from the perspective of single-cell epigenomic profiling (Figure 11, B and C), coincident with previous FACS-based sorting analysis (8). Moreover, the *Il4*^+^ macrophages, which have not been reported in other scRNA-Seq profiling studies, were specifically detected in the neonatal P1 and P10 hearts through our single-cell H3K27ac ChIP-Seq profiling. One reason for this discrepancy may be that previously single-cell RNA studies primarily focused on adult mouse hearts. Additionally, the differences in cell type annotation based on the gene activities delineated by the surrounding enhancer signals and gene expression levels defined by the copy number of its transcripts may contribute to the distinctive level of cellular heterogeneity revealed through single-cell transcriptional and epigenomic profiling.

Consistent with previous studies indicating that P1 hearts possess stronger regeneration capacity than P10 hearts (6), our single-cell ChIP-Seq data offered further insights into the intricate cellular and molecular mechanisms underlying these disparities. First, we characterized the distribution dynamics of the macrophage subtypes with disparate functions across different regions and at distinct time points following MI in P1 and P10 hearts. This exploration revealed a notable prevalence of reparative subpopulations (*Arg1*^+^ macrophages), rather than inflammatory subpopulations (*S100a9*^+^*Ly6c*^+^), within the IZ of P1 hearts 3 days after MI injury, in contrast with the scenario observed in P10-MI_3D hearts, where inflammatory subpopulations were more prominent. The dynamics of MPC mobilization in the IZ partially contribute to the differential regenerative activities between P1 and P10 hearts. Second, cells from hearts after P1 MI exhibited higher enhancer activities associated with marker genes linked to wound healing, even within the same subpopulation. Conversely, stronger enhancer signals adjacent to proinflammatory marker genes were evident in cells from P10-MI hearts than in those from P1-MI hearts (Figure 2A and Figure 5D). These findings reveal previously unrecognized epigenetic heterogeneities within single-cell subclusters and suggest that these epigenetic heterogeneities might constitute the molecular basis underlying regenerative and nonregenerative responses in P1 and P10 hearts, respectively, following MI. Globally, the fractional increases in most populations after MI seem to be very minor, except for *Arg1*^+^ C4 and *S100a9*^+^*Ly6c*^+^ C6, suggesting that the starting composition and the total number of MPCs may dictate cardiac regenerative potential. Consequently, the most notable disparity observed in the steady-state P1 and P10 hearts in our dataset is the C1 population, which is unique to P10 hearts and shares marker genes with C0. However, validating the function of C1 on P10 cardiac regeneration poses a challenge, as it is difficult to specifically perturb C1 without disturbing C0 cells.

While *S100a9* and *Cxcr2* are highly expressed in neutrophils, the *Cxcr2*^+^*S100a9*^+^ subcluster was largely negative for *Ly6g* and unaffected by neutrophil depletion in P10-MI_3D hearts, indicating its monocyte identity (Figure 8, F and G and Supplemental Figure 3). This subcluster resembled the *S100a9*^hi^*Ly6c*^hi^ monocytes characterized in injured kidneys by Yao et al. (61), which was also been detected in a recent paper in adult hearts after MI during our paper under revision (62). Targeting the *S100a9*^+^*Ly6c*^+^ subpopulation through CXCR2 inhibition yielded a reduction in the accumulation of proinflammatory C6 cells in the IZ of post-MI P10 hearts, ultimately resulting in a significant improvement in myocardial outcomes after injury.

Although CXCR2 is essential for neutrophil trafficking (63), we found that the enhanced cardiac repair capacity from CXCR2 inhibition was blocked by neutrophil depletion (Figure 8). This suggests that neutrophil depletion results in poor cardiac repair outcomes after MI, overshadowing the positive effects of *S100a9*^+^*Ly6c*^+^ cell depletion. Additionally, intramyocardial injection of FACS-sorted *S100a9*^+^*Ly6c*^+^ C6 IMos into P5 hearts after MI resulted in decreased cardiac reparative capacity (Supplemental Figure 7), confirming the detrimental role of *S100a9*^+^*Ly6c*^+^ C6 cells in cardiac repair after injury. Altogether, the beneficial effects of CXCR2 blockade on cardiac repair are more likely due to the depletion of *S100a9*^+^*Ly6c*^+^ C6 cells rather than reduced neutrophil recruitment.

Although *Arg1*^+^ C4 cells exist in both P1, P10, and adult hearts after MI, the degree of fractional increase is more striking in P1 than in P10 hearts (Supplemental Figure 4, H and I). Moreover, we observed the highest enhancer activities of wound healing–related genes in P1 hearts (Figure 11K). Therefore, the differences in fraction and gene activity of the *Arg1*^+^ C4 population in P1 hearts may result in a less inflammatory microenvironment that somehow impedes the generation of inflammatory *S100a9*^+^*Ly6c*^+^ C6 cells in P1 hearts compared with P10 and adult hearts, consistent with the previous studies showing that the early neonate mammalian immune system has compromised proinflammatory capacity (64). Further, the suppressed expansion of *S100a9*^+^*Ly6c*^+^ C6 cells in P1-MI hearts compared with P10-MI hearts may result from differences in the sources of monocytes, which may possess differential differentiation potential. In P1 hearts, monocytes can originate from fetal liver, spleen, and bone marrow, while in P10 hearts, they are predominantly derived from bone marrow (65).

The differential preference for the generation of monocyte-derived *Arg1*^+^ C4 or *S100a9*^+^*Ly6c*^+^ C6 in P1 and P10 hearts after injury also suggests that the monocytes may follow distinct trajectories at these time points. Specifically, we speculate that monocyte differentiation into both *Arg1*^+^ C4 and *S100a9*^+^*Ly6c*^+^ C6 in P10 hearts, while only C4 is specifically generated from monocytes in P1 hearts after injury. However, these hypotheses demand further experimental validation.

## Methods

### Sex as a biological variable.

Our study utilized both male and female mice for neonatal MI, as sex was not considered as a biological variable. However, only adult male mice were used for adult MI due to their exhibited lower variability in phenotype. Therefore, the findings are expected to be relevant to both males and females, although no experiments were performed to test for differences between the sexes.

### Statistics.

The statistical analysis was performed on R and all values were shown as mean±SEM. Two groups were compared using either paired or unpaired, 2-tailed *t* tests. Multiple comparisons were made using 1-way ANOVA followed by post hoc Dunnett’s test, Scheffe’s test and LSD test, or Kruskal-Wallis H test followed by post hoc Dunn’s test, as indicated in the figure legends. For all statistical tests, the 0.05 P value was considered statistically significant.

### Study approval.

All animal procedures were conducted in accordance with the local regulations and approved by the Institutional Animal Care and Use Committee of Southern Medical University (SMUL2023045).

### Data availability.

The single-cell CoBATCH dataset in neonatal and adult mouse generated in this study have been deposited in the GEO database at accession code GSE225615 and GSE263798 and the Genome Sequence Archive database in BIG Data Center under accession numbers PRJCA028948 (https://ngdc.cncb.ac.cn/search/specific?db=bioproject&q=PRJCA028948). The single-cell RNA-Seq data are available in the GEO under accession number in GSE235275. All custom code used in this study is available from the corresponding author upon reasonable request. A Supporting Data Values file is available online as supplemental material.

## Author contributions

SA designed and conceived the study. MF and YZ performed the bioinformatic analyses. SJ, LX, XL, and YL conducted the experiments. SA and MF wrote the paper with input from all other authors. All authors participated in data discussion and interpretation.

## Supplementary Material

Supplemental data

Supplemental tables 1-22

Supporting data values

## Version 1 08/27/2024

In-Press Preview

## Version 2 10/01/2024

Electronic publication
