# Supplementary material for: Single-cell multiomic analysis identifies macrophage subpopulations in promoting cardiac repair
Source: J Clin Invest. 2024 Aug 27;134(19):e175297. doi: 10.1172/JCI175297 (PMC11444165; doi:10.1172/JCI175297)
Supplement: Supplemental data [file jci-134-175297-s111.pdf]

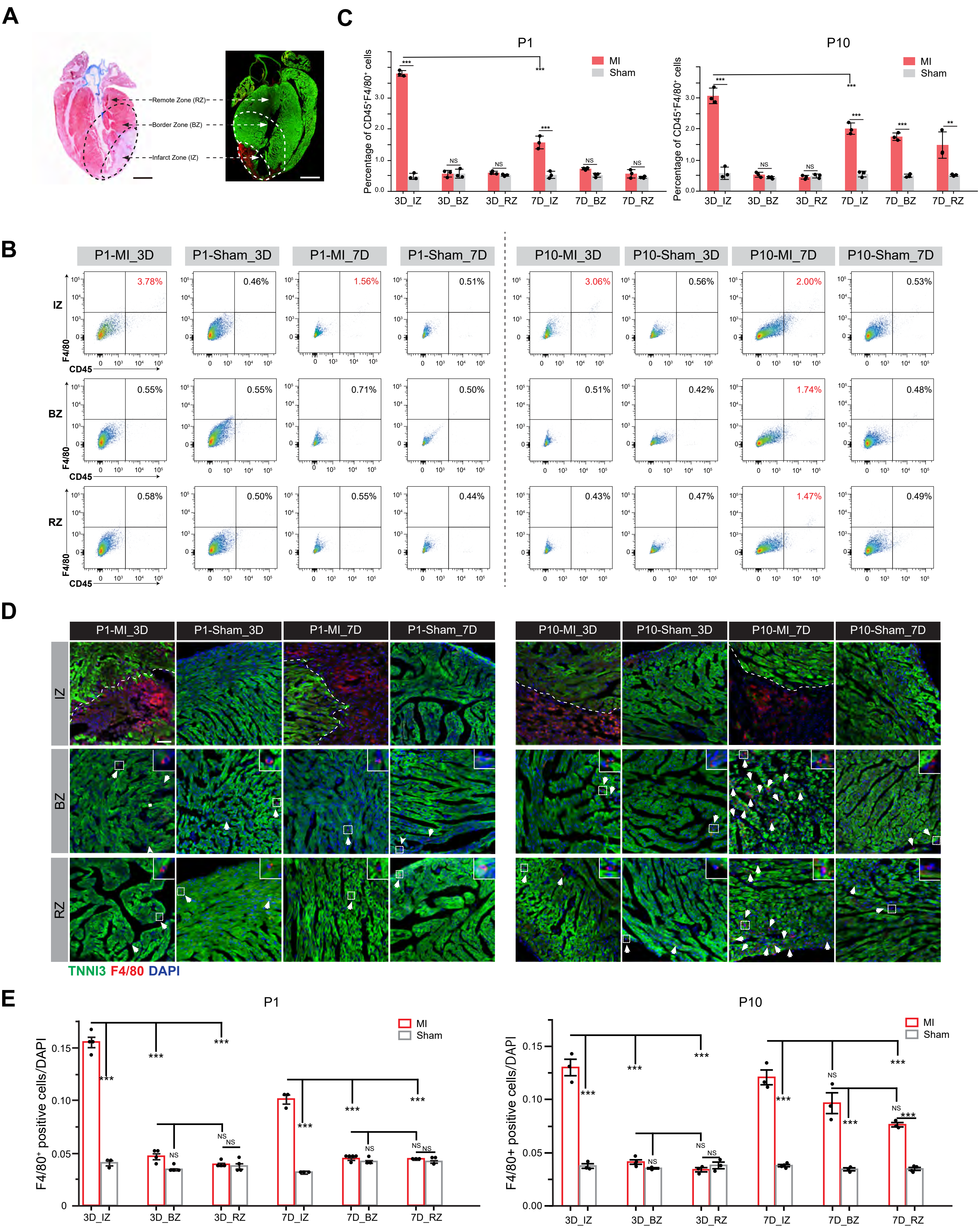

A
Remote Zone (RZ)
Border Zone (BZ)
Infarct Zone (IZ)
C
P1
P10
MI
Sham
Percentage of CD45⁺F4/80⁺ cells
3D_IZ
3D_BZ
3D_RZ
7D_IZ
7D_BZ
7D_RZ
B
P1-MI_3D
P1-Sham_3D
P1-MI_7D
P1-Sham_7D
P10-MI_3D
P10-Sham_3D
P10-MI_7D
P10-Sham_7D
IZ
BZ
RZ
F4/80
CD45
3.78%
0.46%
1.56%
0.51%
3.06%
0.56%
2.00%
0.53%
0.55%
0.55%
0.71%
0.50%
0.51%
0.42%
1.74%
0.48%
0.58%
0.50%
0.55%
0.44%
0.43%
0.47%
1.47%
0.49%
D
TNNI3 F4/80 DAPI
E
F4/80⁺ positive cells/DAPI
F4/80+ positive cells/DAPI

Supplemental Figure 1. Macrophages/monocytes abundance and localization in hearts following MI of P1 and P10 hearts. (**A**) Graphic representation of spatial regions of the injured hearts. The hearts were collected at 3 days post P10 MI and suffered Masson's trichrome staining (left) and immunofluorescence staining (right). TNNI3 (Green) and FSP1 (Red) label cardiomyocytes and myofibroblasts, respectively. Scale bar equals 1,000 μM. (**B**) Representative flow cytometry analysis of cardiac $CD45^+F4/80^+$ macrophages/-monocytes at 3 and 7 days post P1 (left panel) and P10 (right panel) MI/Sham at infarct zone (IZ), border zone (BZ), and remote zone (RZ) of mouse hearts. n=3 per experimental group, and 3-4 mouse hearts were combined for one FACS experiment. (**C**) Quantification of the percentage of $CD45^+F4/80^+$ macrophages/monocytes in (**B**). (**D**) Representative immunostaining for F4/80 (Red), TNNI3 (Green), and DAPI to visualize macrophages/monocytes in IZ (top), BZ (middle) and RZ (bottom) of mouse hearts at 3 and 7 days after P1 (left panel) and P10 (right panel) MI/Sham. Scale bar equals 50 μM. (**E**) Quantification of the percentage of $F4/80^+$ macrophages/monocytes in (**D**). n=3-5 mice per experimental group. The *P*-value was determined by One-way ANOVA with post hoc scheffe's test (**C** and **E**). Data represent mean ± SEM. NS, not significant; $*P < 0.05$; $**P < 0.01$; $***P < 0.001$.

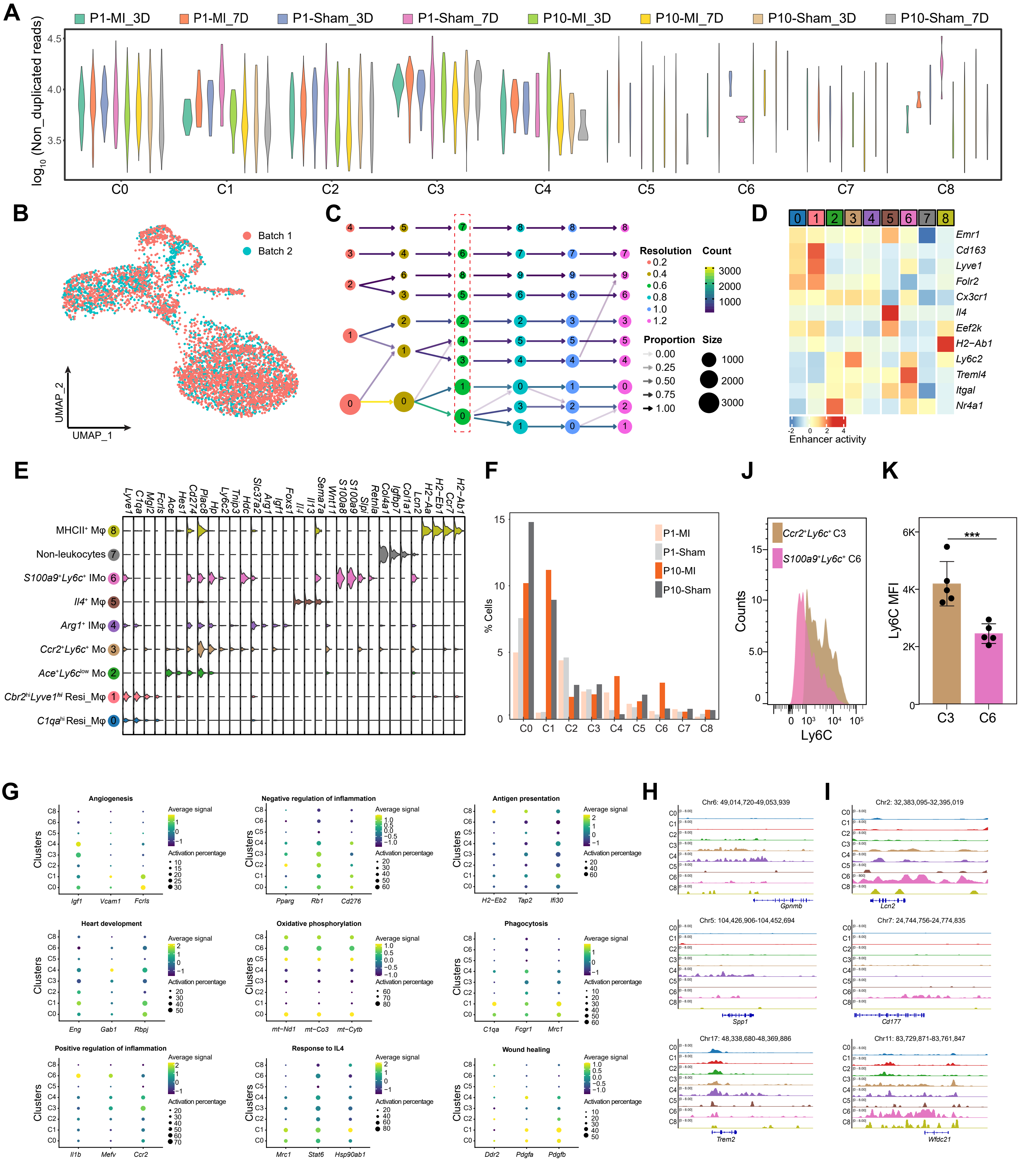

A
P1-MI_3D
P1-MI_7D
P1-Sham_3D
P1-Sham_7D
P10-MI_3D
P10-MI_7D
P10-Sham_3D
P10-Sham_7D
log10 (Non_duplicated reads)
B
Batch 1
Batch 2
UMAP_1
UMAP_2
C
Resolution
Count
Proportion
Size
D
Enhancer activity
E
MHCII+ Mφ
Non-leukocytes
S100a9+Ly6c+ IMo
Il4+ Mφ
Arg1+ IMφ
Ccr2+Ly6c+ Mo
Ace+Ly6clow Mo
Cbr2hiLyve1hi Resi_Mφ
C1qahi Resi_Mφ
F
% Cells
P1-MI
P1-Sham
P10-MI
P10-Sham
J
Ccr2+Ly6c+ C3
S100a9+Ly6c+ C6
Counts
Ly6C
K
Ly6C MFI
***
G
Angiogenesis
Negative regulation of inflammation
Antigen presentation
Heart development
Oxidative phosphorylation
Phagocytosis
Positive regulation of inflammation
Response to IL4
Wound healing
Average signal
Activation percentage
Clusters
H
Chr6: 49,014,720-49,053,939
Gpnmb
Chr5: 104,426,906-104,452,694
Spp1
Chr17: 48,338,680-48,369,886
Trem2
I
Chr2: 32,383,095-32,395,019
Lcn2
Chr7: 24,744,756-24,774,835
Cd177
Chr11: 83,729,871-83,761,847
Wfdc21

Supplemental Figure 2. Enhancer activities of marker genes in each cluster.
(**A**) Violin plot showing the number of deduplicated reads in each cell across subclusters in the H3K27ac CoBATCH dataset indicating comparable distributions. (**B**) UMAP embedding of the H3K27ac CoBATCH dataset colored by two batches. (**C**) Clustering tree showing cluster results for resolution parameters ranging from 0.2 to 1.2 in Seurat FindClusters function. The red dashed box highlights the resolution 0.6 used in this study. (**D**) Heatmap showing the row-normalized H3K27ac ChIP-seq signals around typical monocyte and macrophage-relevant markers. (**E**) Violin plots displaying the normalized H3K27ac ChIP-seq signals around representative celltype-specific gene loci. (**F**) Quantification of the percentage of cells in each cluster. (**G**) Dot plot showing enhancer activities of representative genes related to GO functions in **Figure 1E** in each cluster. (**H** and **I**) Genome browser tracks of celltype-specific H3K27ac ChIP-seq signals in C4 (**H**) and C6 (**I**). (**J**) Representative histograms showing the expression of Ly6C in C3 and C6. (**K**) Quantification of mean fluorescence intensity (MFI) of Ly6C in (**J**). n=5 mice per experimental group. The *P*-value was determined by unpaired 2-tailed Student's t-test. Data represent mean ± SEM. *$P < 0.05$; **$P < 0.01$; ***$P < 0.001$.

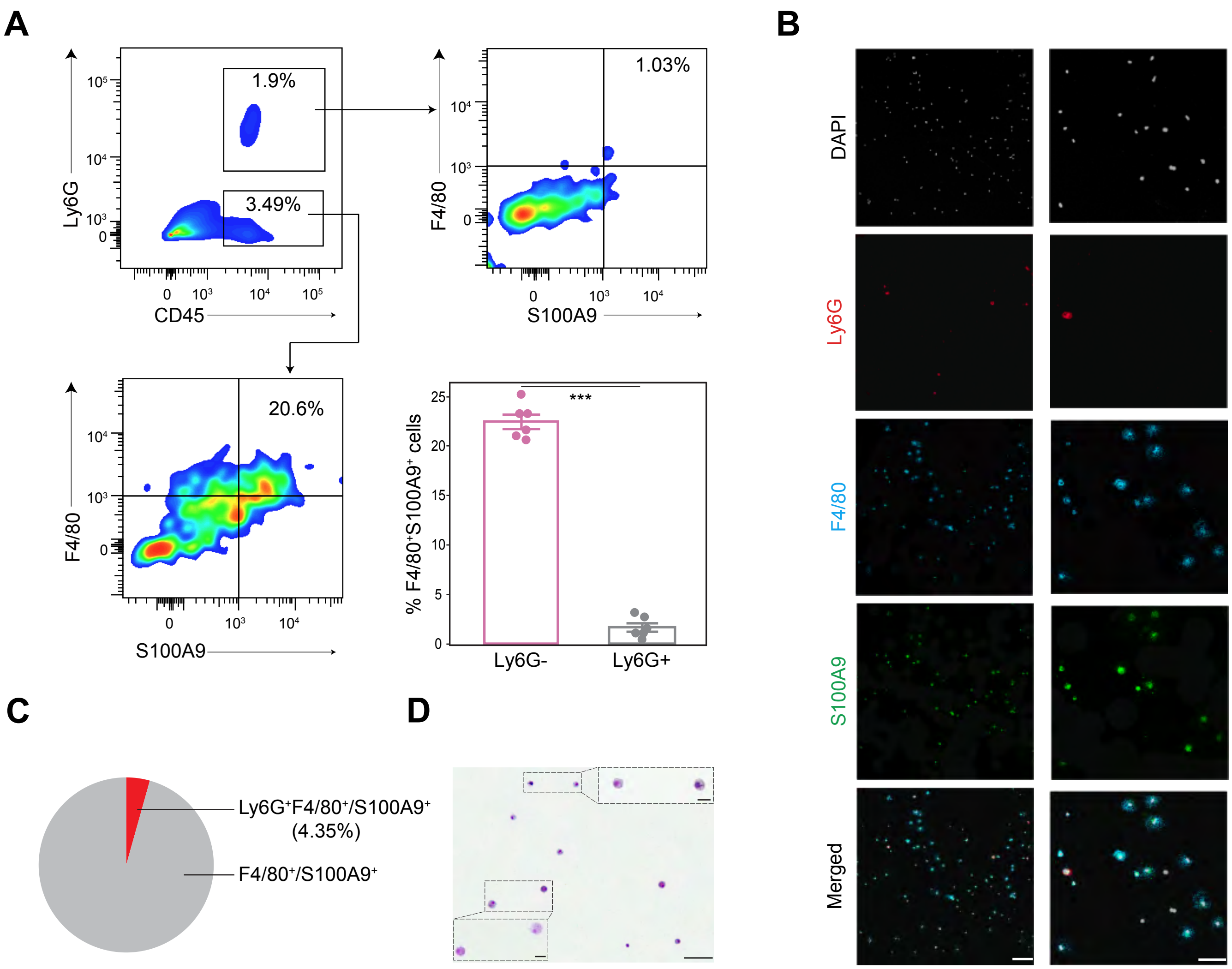

Supplemental Figure 3. Characterization of the identity of *S100a9⁺Ly6c⁺* C6 cells.
(**A**) Representative flow cytometry plot and quantification analysis of the percentage of F4/80⁺S100A9⁺ cells within Ly6G negative and Ly6G positive leukocyte populations. (**B**) Representative immunostaining for DAPI (white), Ly6G (red), F4/80 (blue) and S100A9 (green) of the FACS-sorted C6 cell. The scale bars equals 200 μM (left) and 100 μM (right), respectively. (**C**) Quantification of the number of Ly6G⁺ C6 cells in (**B**). (**D**) Representative image displaying the Giemsa staining of the FACS-sorted CD45⁺F4/80⁺S100A9⁺ C6 cells. Scale bar equals 100 μM and the experiments were repeated for three times. n=6 mice per experimental group in (**A**). The *P*-value was determined by paired 2-tailed Student's t-test. Data represent mean ± SEM. ****P* < 0.001.

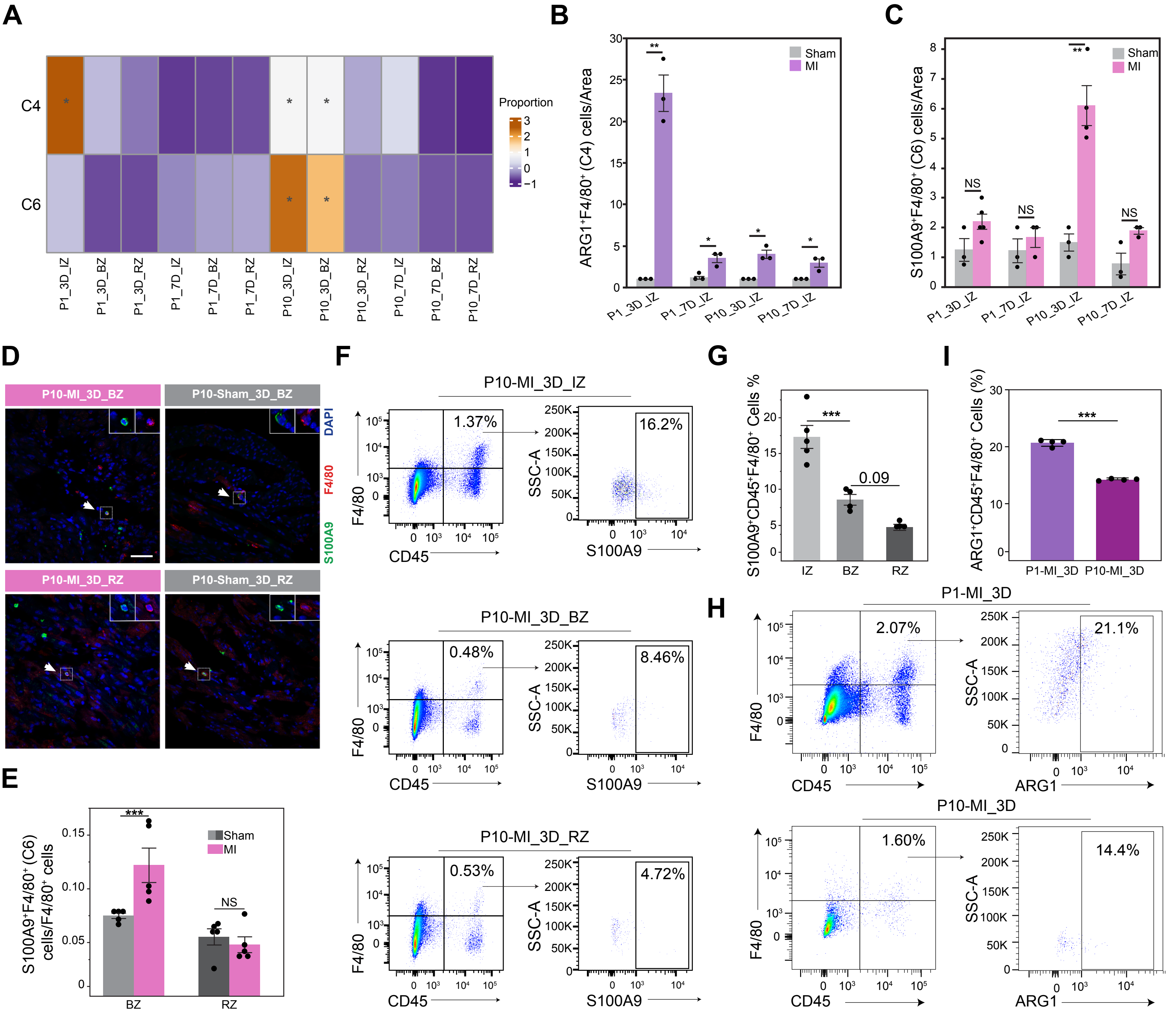

Supplemental Figure 4. Characterization of the abundance of C4 and C6 cells in P1 and P10 hearts post MI.
(**A**) Heatmap displaying the distribution of C4 and C6 cells in distinct regions of P1/P10 MI hearts. The color bar represents values normalized by z-score for each row. Groups with normalized proportion > 0.5 were marked as an asterisk. (**B** and **C**) Quantification of the absolute number of $ARG1^+F4/80^+$ C4 and $S100A9^+F4/80^+$ C6 cells per unit area in **Figure 2B** (**B**) and **2D** (**C**). The unit area indicates 86463 μM^2. (**D** and **E**) Representative immunostaining (**D**) and quantification (**E**) for F4/80 (Red), S100A9 (Green) and DAPI to visualize C6 cells at the BZ and RZ in mouse hearts 3 days after P10 MI. Scale bar equals 50 μM. (**F** and **G**) Flow cytometry analysis (**F**) and quantification (**G**) of the percentage of C6 cells at the IZ, BZ and RZ in mouse hearts 3 days after P10 MI. (**H** and **I**) Flow cytometry analysis (**H**) and quantification (**I**) of the percentage of C4 cells at 3 days post P1 and P10 MI. n=3-7 mice per experimental group in (**B**-**I**). The *P*-value was determined by paired 2-tailed Student's t-test (**B**, **C**, **E** and **I**) and One-way ANOVA with post hoc Dunnett's test (**G**). Data represent mean ± SEM. NS, not significant; *$P < 0.05$; **$P < 0.01$; ***$P < 0.001$.

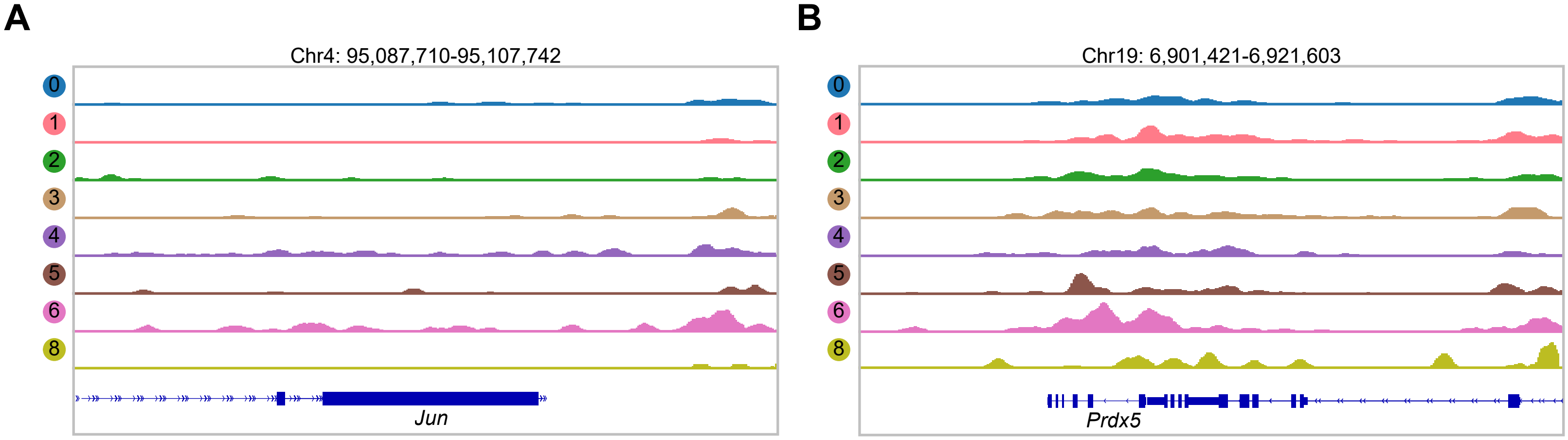

Supplemental Figure 5. Cluster-specific enhancer signals.
(**A** and **B**) Genome browser visualization of H3K27ac ChIP-seq signals around *Jun* (**A**) and *Prdx5* (**B**) CCRNs in different cell subpopulations.

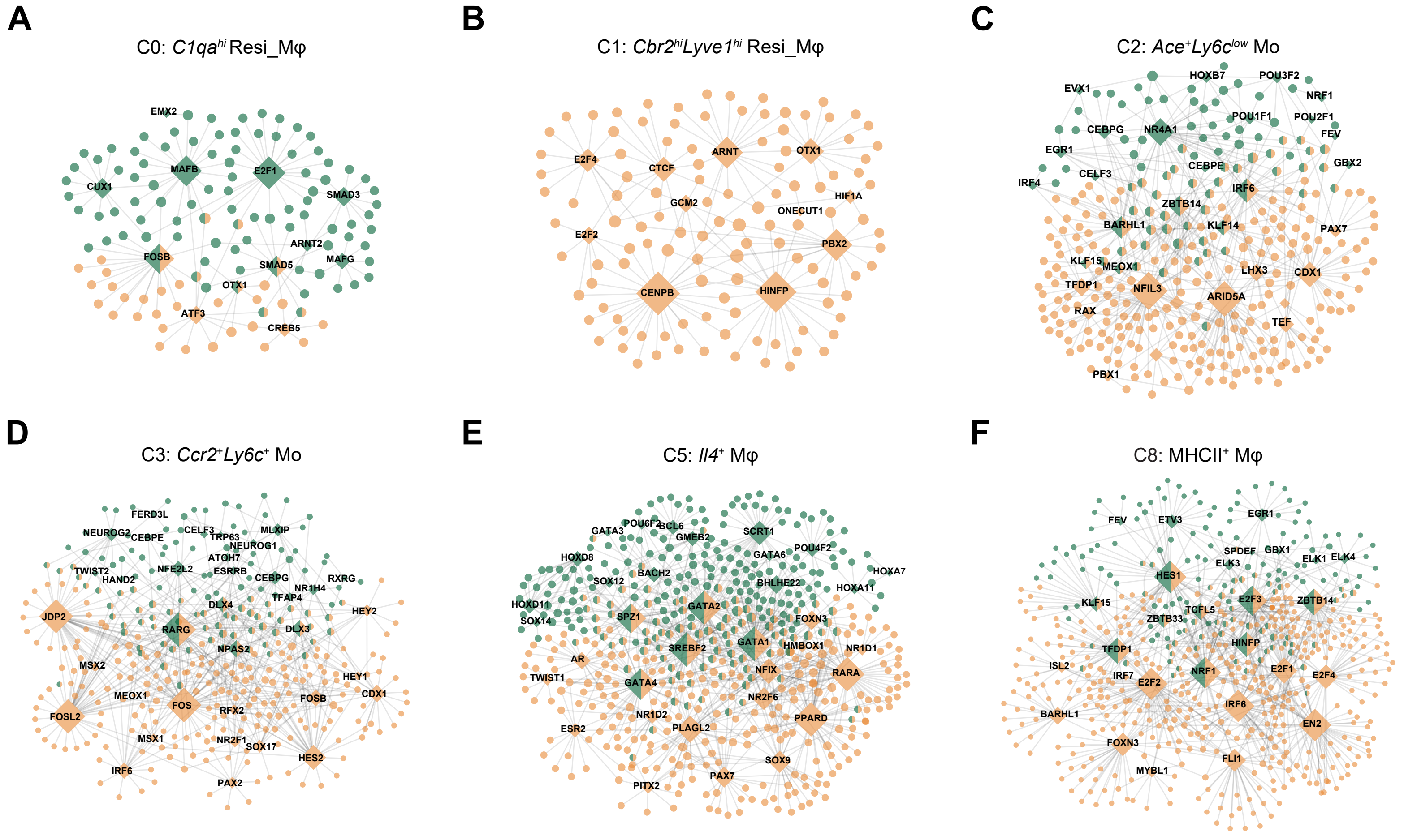

Supplemental Figure 6. Differential TF-TG regulatory networks in macrophage/monocyte subtypes between P1 and P10. (**A**-**F**) TF regulatory network showing specific and shared key TFs and their target genes (TGs) between P1 (green) and P10 (orange) hearts in C0 (**A**), C1 (**B**), C2 (**C**), C3 (**D**), C5 (**E**), and C8 (**F**). The edges indicate TF-TG pairs, and the size of the dot indicates the number of nodes in the network. The green and orange bicolor represent TFs or TGs shared by P1 and P10 cells.

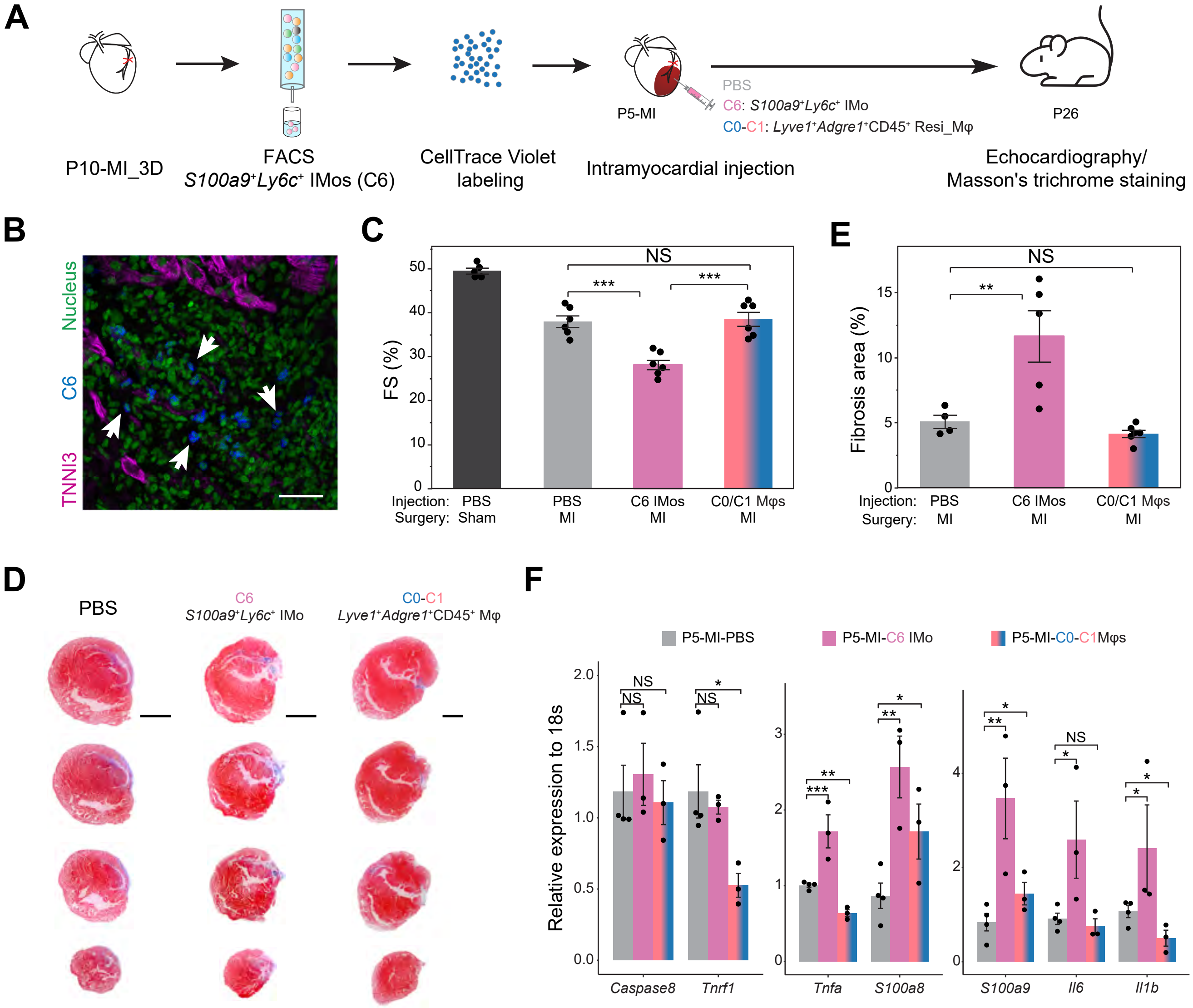

Supplemental Figure 7. Intramyocardial injection of *S100a9*⁺*Ly6c*⁺ IMos exacerbates cardiac injury after P5 MI.
(**A**) Schematic illustration depicting the experimental design for reintroducing *S100a9*⁺*Ly6c*⁺ C6 IMos and *Lyve1*⁺*Adgre1*⁺CD45⁺ C0/C1 Mφs. (**B**) Representative images of immunofluorescence analysis of the FACS-sorted cells labeled with CellTrace Violet in the P5-MI_3D hearts. The nuclei were stained with Helix NP™ Green and the scale bar equals 50 μM. (**C**) Echocardiographic measurements of heart function 3 weeks post P5 MI. (**D**) Masson trichrome staining of cross-sections from hearts injected with *S100a9*⁺*Ly6c*⁺ C6 IMos, *Lyve1*⁺*Adgre1*⁺CD45⁺ C0/C1 Mφs and PBS 3 weeks post P5 MI. Scale bar equals 200 μM. (**E**) Quantification analysis of the fibrosis area in (**D**). (**F**) Quantification of the relative expression of representative pro-inflammatory genes in P5-MI hearts 3 days post injection of PBS, FACS-sorted *S100a9*⁺*Ly6c*⁺ C6 cells, and FACS-sorted *Lyve1*⁺*Adgre1*⁺CD45⁺ C0/C1 macrophages. n=3-6 mice per experimental group. The *P*-value was determined by One-way ANOVA with post hoc Scheffe's test (**C**), with post hoc Dunnett's test (**E**), or with post hoc Conover-Iman test (**F**). Data represent mean ± SEM. $^{*}P < 0.05$; $^{**}P < 0.01$; $^{***}P < 0.001$.

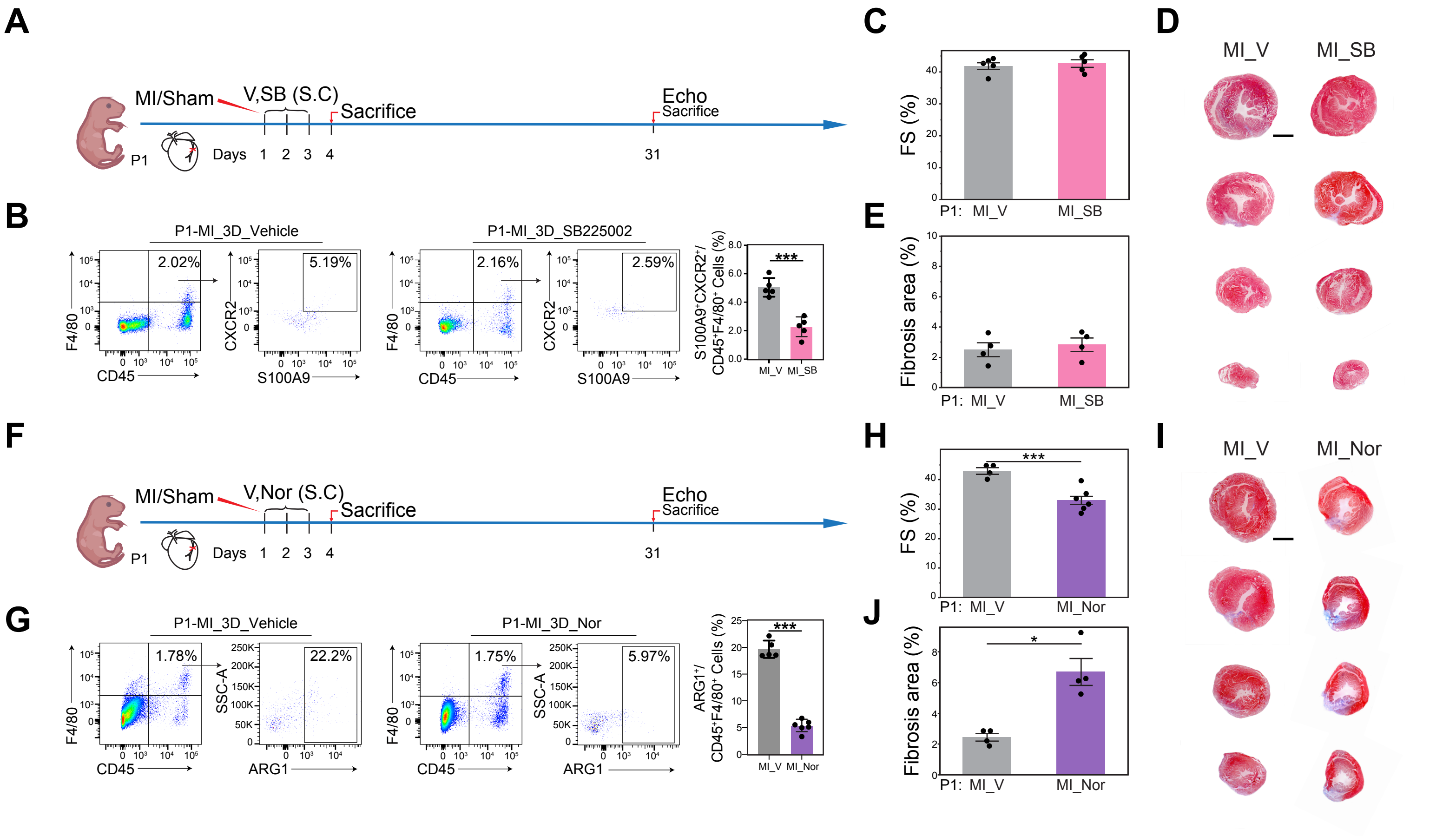

Supplemental Figure 8. Cardiac output after targeting *S100a9*⁺*Ly6c*⁺ IMos and *Arg1*⁺ IMφs in hearts post P1 MI.
(**A**) Schematic illustration depicting the experimental design for targeting *S100a9*⁺*Ly6c*⁺ IMos in P1 MI hearts. (**B**) Flow cytometry showing the percentage of S100A9⁺CXCR2⁺ IMos in hearts injected with SB225002 (SB) or Vehicle (V) in (**A**). (**C**) Echocardiographic measurements of heart function at one month post P1 MI in each treatment group in (**A**). (**D**) Masson trichrome staining of cross-sections from hearts injected with SB225002 (SB) or Vehicle (V). Scale bar equals 200 μM. (**E**) Quantitative analysis of the fibrosis area in (**D**). (**F**) Schematic illustration depicting the experimental design for targeting *Arg1*⁺ IMφs in P1 MI hearts. (**G**) Flow cytometry showing the percentage of ARG1⁺ IMφs in hearts injected with nor-NOHA monoacetate (Nor) or Vehicle (V) in (**F**). (**H**) Echocardiographic measurements of heart function at one month post P1 MI in each treatment group in (**F**). (**I**) Masson trichrome staining of cross-sections from hearts injected with nor-NOHA monoacetate (Nor) or Vehicle (V). Scale bar equals 200 μM. (**J**) Quantitative analysis of the fibrosis area in (**I**). n=4-6 mice per experimental group. The *P*-value was determined by paired (**B**, **C**, **E**, **J**) and unpaired (**G**, **H**) 2-tailed Student's t-test. Data represent mean ± SEM. *$P < 0.05$; **$P < 0.01$; ***$P < 0.001$.

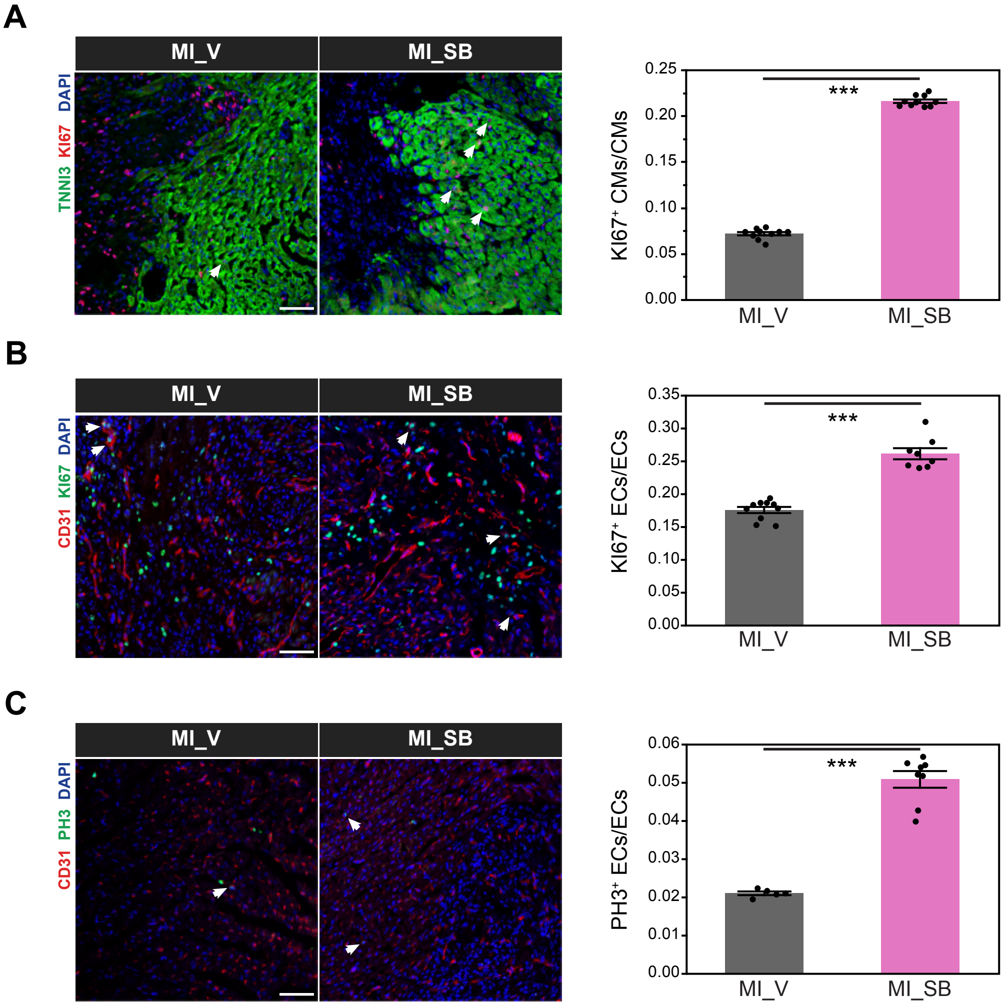

Supplemental Figure 9. Examination of the proliferation states of cardiomyocytes and endothelial cells in P10-MI_3D hearts after CXCR2 blockade.

(**A**) Representative immunostaining and quantification for $KI67^{+}TNNI3^{+}$ proliferative CMs in the BZ of P10-MI_3D hearts injected with SB225002 (SB) or Vehicle (V). (**B** and **C**) Representative immunostaining and quantification for $KI67^{+}CD31^{+}$ (**B**) and $PH3^{+}CD31^{+}$ (**C**) proliferative ECs in the BZ of P10-MI_3D hearts injected with SB225002 (SB) or Vehicle (V). n=5-10 mice per experimental group. Scale bars in (**A**, **B** and **C**) equal 50 μM. The *P*-value was determined by unpaired 2-tailed Student's t-test. Data represent mean ± SEM. **P* < 0.05; ***P* < 0.01; ****P* < 0.001.

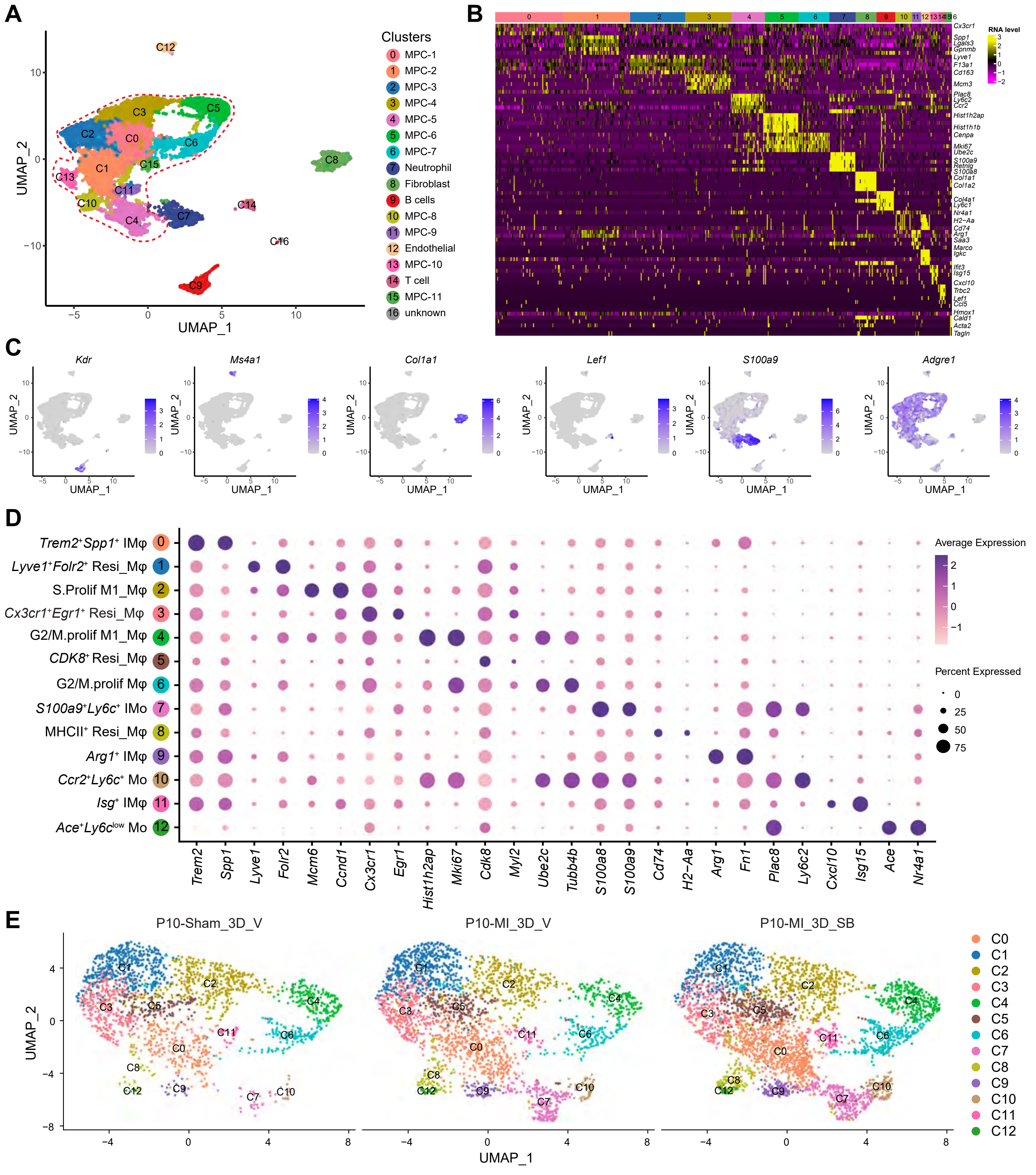

Supplemental Figure 10. Identification of MPC populations and marker gene expression in scRNA-seq data.
(**A**) UMAP plot of a total of 10,546 cells which passed stringent quality control. Clusters 0, 1, 2, 3, 4, 5, 6, 10, 11, 13, and 15 were MPCs and highlighted by red circles. (**B**) Heatmap showing the top 5 genes of individual clusters. (**C**) Feature plots of marker genes used to identify and annotate MPC. (**D**) Dot plot of the representative maker genes of each MPC subcluster. (**E**) UMAP visualization of MPC clusters from three different experimental conditions, annotated by clusters as in **Figure 6B**.

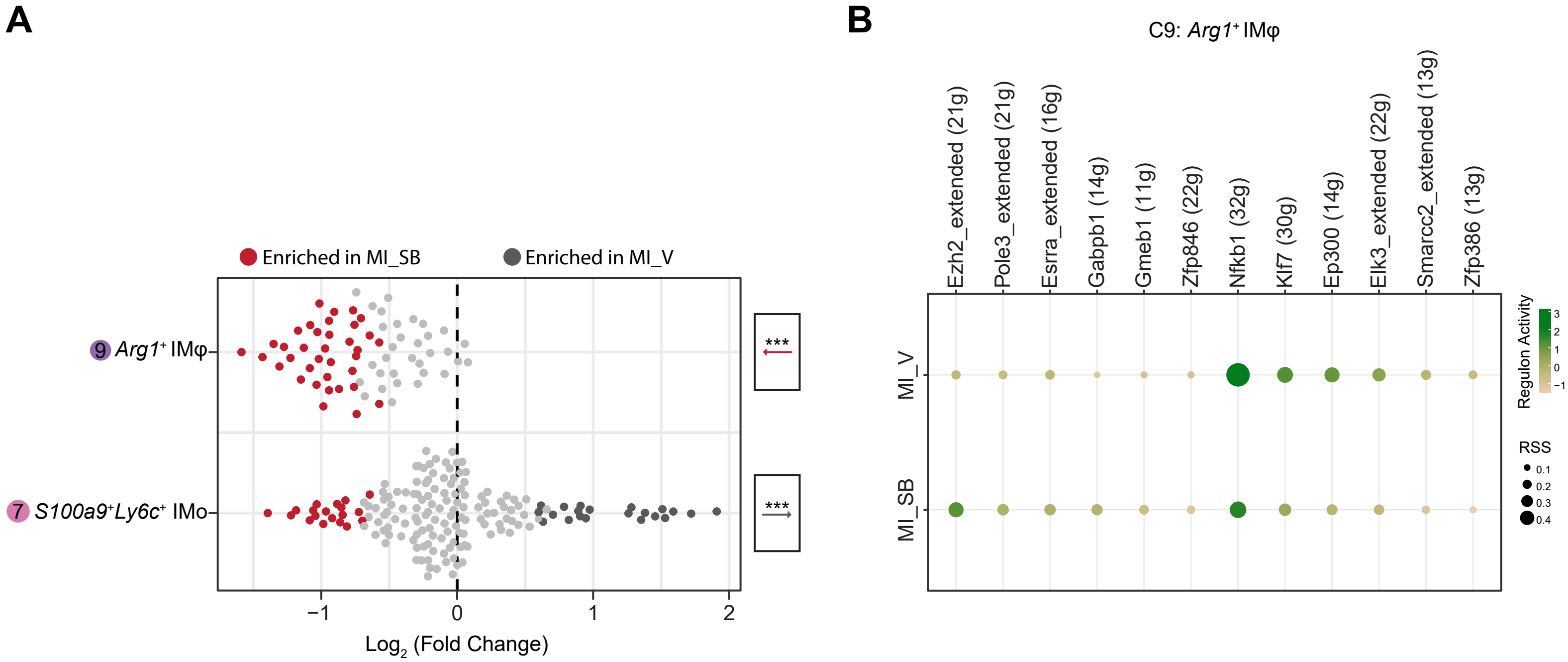

Supplemental Figure 11. Characterization of cell abundance and gene expression after CXCR2 blockade in *Arg1*⁺ C9 and *S100a9*⁺*Ly6c*⁺ C7 scRNA-seq clusters.

(**A**) Beeswarm plot showing the distribution of log fold change in abundance between P10-MI_SB and P10-MI_V groups in *Arg1*⁺ C9 and *S100a9*⁺*Ly6c*⁺ C7 clusters. Differential abundance neighborhoods at FDR 10% are colored. Cells detected as enriched in SB225002 (SB) or Vehicle (V) treated hearts are annotated on the right side. The *P*-value was determined by the Wilcox test. ****P* < 0.001. (**B**) Dotplot displaying the scaled activity scores of regulons for cells from P10-MI_V and P10-MI_SB in C9. The dot size indicates the regulon specificity score (RSS) and the color indicates the Z-score of the Regulon activities.

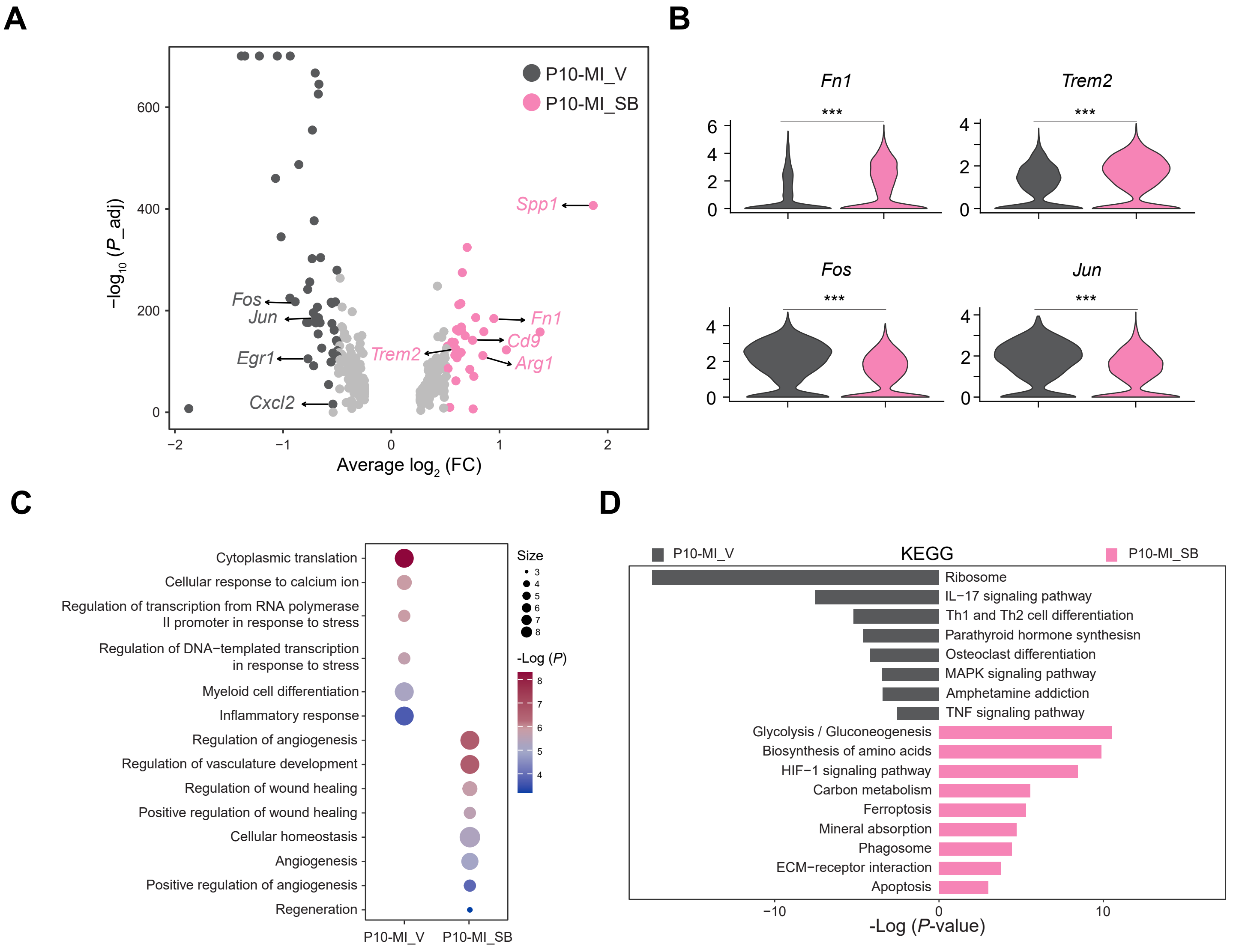

Supplemental Figure 12. CXCR2 blockade reduces the proinflammatory while increasing the reparative responses after MI. (**A** and **B**) Volcano plot showing significantly differentially expressed genes between SB225002 (SB) and Vehicle (V) treated hearts after P10 MI (**A**), as well as the representative gene expression comparison (**B**). The pseudo-bulks of SB225002 (SB) and Vehicle (V) groups were aggregated using single cells. *P*-value was calculated by the Wilcox test. ****P* < 0.001. (**C**) Gene ontology enrichment for differentially expressed genes between SB225002 (SB) and Vehicle (V) treated hearts suffered MI at P10. *P*-value was calculated by the Binomial test. (**D**) KEGG pathway analysis of differentially expressed genes between SB225002 (SB) and Vehicle (V) treated hearts suffered MI at P10. The *P*-value was calculated by Fisher's exact test.

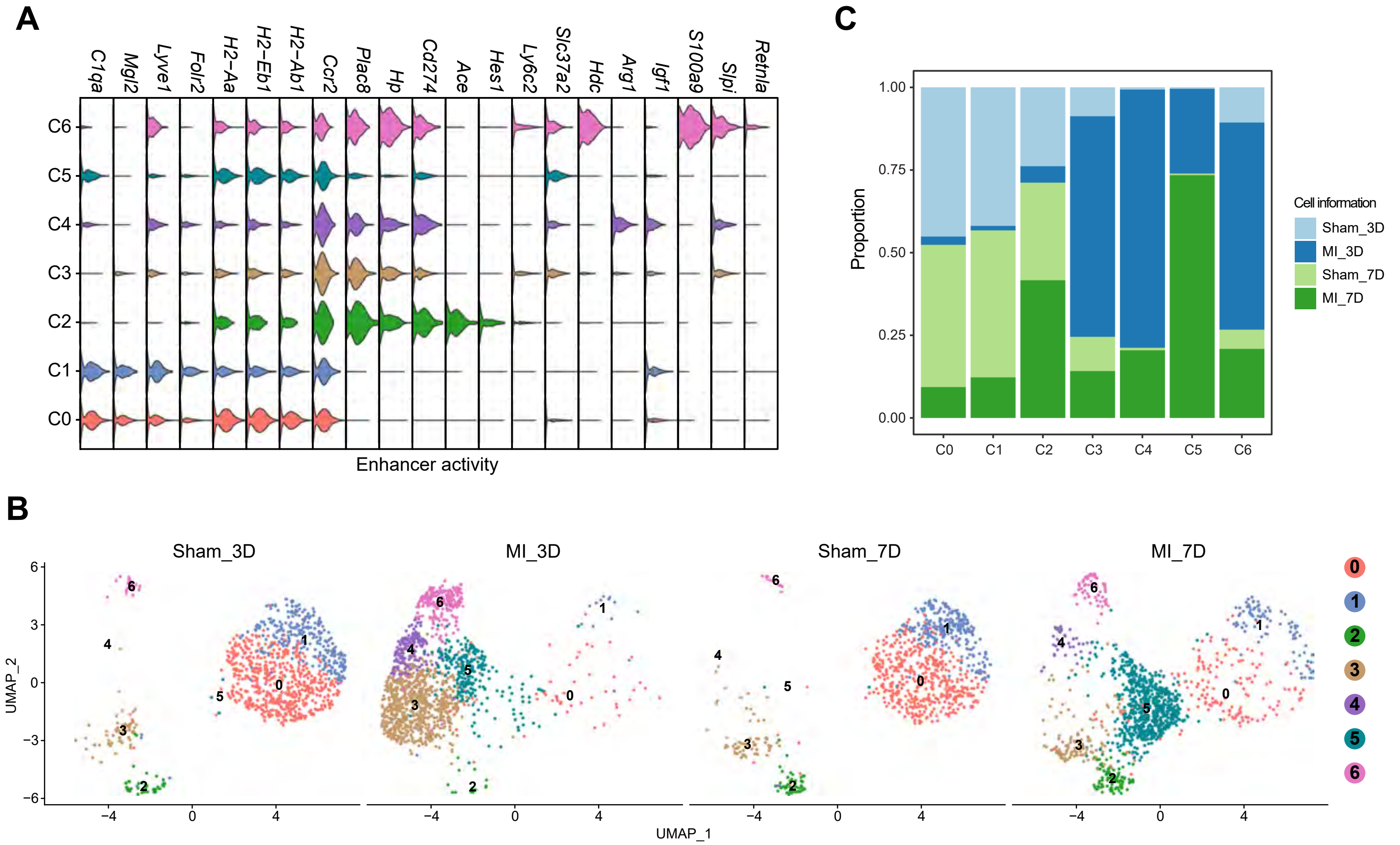

Supplemental Figure 13. Macrophage/monocyte subpopulation composition in adult mouse heart post-MI.
(**A**) Violin plot showing the enhancer activity of representative maker genes in each subcluster. (**B**) UMAP visualization of macrophage/-monocyte clusters from four different experimental conditions, annotated by cluster information as depicted in **Figure 11B**. (**C**) Bar plot showing the cell composition from four different experimental conditions in each subcluster.

Methods

Sex as a biological variable

Our study utilized both male and female mice for neonatal myocardial infarction, as sex was not considered as a biological variable. However, only adult male mice were used for adult myocardial infarction due to their exhibited lower variability in phenotype. Therefore, the findings are expected to be relevant to both males and females, although no experiments were performed to test for differences between the sexes.

The research animals

Wild-type C57BL/6 mice were used for this study. All mice were bred and housed in a 12 h light/dark cycle temperature-controlled room, with ad libitum access to water and food. All animal experiments described in this manuscript have been approved and conducted under the oversight of the Institutional Animal Care and Use Committee of Southern Medical University (SMUL2023045). Timed-pregnant C57BL/6 mice were used to deliver pups for surgical procedures on postnatal day 1 (P1) or 10 (P10). Sex was not determined for neonatal pups.

P1 and P10 myocardial infarction model

Myocardial infarction (MI) surgeries were performed on P1 and P10 mice as previously described (1). Briefly, Neonatal mice were anesthetized by hypothermia on an ice bed for about 5-12 min, lateral thoracotomy was performed and a 7-0 prolene suture was tied through the left anterior descending coronary artery to induce infarction. Sham-operated mice underwent the same procedure without left anterior descending ligation. After surgery, incisions were sutured with a 7-0 nonabsorbable prolene suture, and neonates were allowed to recover under a heat lamp for several minutes.

Adult myocardial infarction

8-week-old C57BL/6J male mice were rotated to undergo Sham or MI surgery. Mice were anesthetized by intraperitoneal treatment of 10 mg/ml sodium pentobarbital at a dose of 70 mg/kg, and then a small-animal respirator was inserted into the mice under anesthesia to assist respiration. In order to get access to the heart, a small skin incision was made in the chest, followed by the removal of muscle layers at the level of the third rib. The rib was sectioned and a small orifice was done in the chest, and a knot was made in the proximal left anterior descending (LAD) using the 8-0 polypropylene suture. Rib, muscle, and skin incisions were sutured with 6-0 sutures. For Sham mice, the coronary artery ligation operation was omitted.

SB225002 treatment

SB225002 (SB) was dissolved in vehicles (2% DMSO+30% PEG 300+5% Tween 80 in PBS), and administered by daily intraperitoneal injection at a dosage of 2.5 mg/kg of body weight after P10 MI for three days. As a control, the same volume of vehicles was injected. Mice were subjected to echocardiography at 1 and 3 weeks after MI. Finally, the hearts were collected for histology analysis.

Neutrophil depletion

Anti-Ly-6G or anti-IgG2a was intraperitoneally injected at a dose of 25 μg into mice at 24 h before P10 MI, and at a dose of 50 μg at day 1-, 2- and 3-days post P10 MI. Finally, the hearts were collected for data analysis.

ARG1 inhibitor treatment

ARG1 inhibitor (nor-NOHA monoacetate) was dissolved in vehicles (10% DMSO+40% PEG 300+5% Tween 80 in saline), and administered by daily subcutaneous injection at a dosage of 5

mg/kg of body weight after P1 MI for six days. As a control, the same volume of vehicles was injected. Mice were subjected to echocardiography at one month after MI. Finally, the hearts were collected for histology analysis.

CCR2 inhibitor treatment

CCR2 inhibitor (RS 504393) was dissolved in vehicles (10% DMSO+10% PEG 300 in saline). To validate the origin of C4 cluster, RS 504393 was subcutaneously injected at a dose of 5 mg/kg of body weight 12 hours before P1 MI and continuously for three days after P1 MI. As a control, the same volume of vehicle was injected. Mice were subjected to FACS at 3 days after P1 MI. For the validation of the origin of C1, P7 mice were subcutaneously injected with RS 504393 at a dose of 5 mg/kg of body weight for ten consecutive days. As a control, the same volume of vehicle was injected. Cardiac cells were dissociated and subjected to FACS at P15.

Giemsa staining

$S100A9^{+}F4/80^{+}CD45^{+}$ C6 IMs were sorted by FACS. The cells were fixed by cold methanol and stained with Giemsa buffers (Baso BA4219) at RT for 20 min. The slides were imaged by Zeiss Axio Vert.A 1.

Intramyocardial Injection of C6 IMs

The $CD45^{+}F4/80^{+}S100A9^{+}$ C6 cells were collected from the P10-MI_3D hearts by FACS and labeled by the CellTrace Violet (Invitrogen) according to the manufacturer's instructions. 20,000 labeled cells were resuspended with 20 µl PBS. P5 C57BL/6J pups were anesthetized in ice, and then the heart was exposed by left-sided open thoracotomy, myocardial infarction was induced by permanent ligation of the proximal left anterior descending (LAD), and then the 20 µl cell

suspension was injected into the myocardium below the ligation site. Control groups were injected with 20 µl PBS. Finally, the rib, muscle, and skin incisions were sutured.

RNA extraction and RT-qPCR

Total RNA was extracted from all samples using Trizol (Invitrogen) according to the manufacturer's instruction. Briefly, 0.4 mL of TRIzol Reagent was used to lyse the cells, followed by the addition of 80 µl of phenol-chloroform to facilitate phase separation. RNA was then purified by centrifugation and washed with 75% RNase-free ethanol.

For reverse transcription (RT), 500 ng RNA was reverse transcribed using cDNA synthesis kit (Vazyme R333-01). A 10 µl RT mix was incubated at 50 ℃ for 1 h and 85 ℃ for 5 s after genomic DNA removal. The primers used for RT-qPCR analysis are listed in Supplemental Table 22.

Cardiac cell isolation and macrophage enrichment by flow cytometry

The hearts from MI and Sham groups were collected and manually divided into infarct, border, and remote zone. Samples of the same zone from 3-5 mouse hearts were pooled together. The heart tissues were minced into 1-3 mm pieces on ice with sterilized scissors in 2 mL tubes containing 1 mL 5% FBS (with 10 mM sodium butyrate) and washed twice with 1 mL cold HBSS (with 10 mM sodium butyrate) to remove excessive blood. Next, tissues were individually digested with 1 mL 0.075% Collagenase II (Sigma-Aldrich, #C6885) at 37°C for 8-15 min in the rotator with 40 rpm, and cells were collected by centrifugation at 500 g for 5 min at 4°C. This digestion step was repeated for 3-4 times until the tissue was fully digested. To get rid of blood cells, cells were suspended with 1 mL RBC (erythrocyte lysis buffer, 155 mM NH_4Cl, 10 mM $KHCO_3$ and 0.01 mM EDTA in sterilized ddH_2O) and incubated on ice for 3 min. RBC buffer was removed by

centrifugation at 500 g for 5 min at 4°C and the cells were then resuspended with 1 mL 5% FBS (10 mM sodium butyrate).

Cell suspensions were stained in 5% FBS (10 mM sodium butyrate) with fluorochrome-labeled antibodies (Supplemental Table 21): anti-F4/80-APC (Biolegend, 123116, 1:100); anti-CD45-PE-Cy7 (Biolegend, 103114, 1:400); 7-AAD (Invitrogen, 00699350, 1:100). Cells were sorted on a FACSAriaIII. FSC-W vs FSC-A was used to exclude doublets and dead cells were removed by 7-AAD. Macrophages were identified as CD45 and F4/80 double-positive cells. The gating strategy for those markers was set based on the background auto-fluorescence measured in unstained controls. The dissociated cells were bound with activated Concanavalin A coated magnetic beads (Con-A beads) for 15 min at RT. Then cells were cross-linked with 0.25% formaldehyde at 4°C for 5 min and quenched by the addition of 35 mM glycine at 4°C for 5 min followed by methyl alcohol fixation. Samples were stored at 80°C for later use.

Low input *in situ* ChIP

Adequate cells were taken out from -80°C and washed thrice with 0.1% BSA/PBS (with 10 mM sodium butyrate) for 5 min each time. Cells were resuspended with 80 µl Antibody Buffer (mix 4 µL 0.5 M EDTA with 1 mL 0.01% Digitonin-Wash Buffer (Dig-Wash Buffer) supplemented with cocktail, 10 mM sodium butyrate and 0.05% Triton X-100) containing 0.4 µg primary antibody. The mixture was incubated at 4°C for 4 h in a rotator with 30 rpm. Cells were then washed twice with 180 µL Dig-Wash Buffer. The secondary antibody was incubated in the same buffer and condition except for the duration of 10 min. After washing with Dig-Wash Buffer twice, cells were suspended with 80 µl Dig-Wash Buffer (with 0.05% Triton-X, cocktail, and 10 mM sodium butyrate) containing 0.83 µM PAT-MEA/B at 4°C for 1 h. The free PAT-MEA/B were then dislodged with 180 µL Dig-Wash Buffer containing 0.05% Triton X-100 three times at 5 min each

time. The reaction was activated by suspending the cells with 10 μL cold Reaction Buffer (10 mM TAPS-NaOH pH 8.3, 5 mM $MgCl_2$, 10% DMF, 0.05% Triton-X, 0.01% Digtonin and supplemented with cocktail, 10 mM sodium butyrate), followed by gently flicking and incubating at 37°C for 1 h in the Eppendorf ThermoMixer with 300 rpm. The reaction was gently mixed once after 30 min incubation and stopped by the addition of 8 μl 50 mM EDTA followed by further incubation at 4°C for 10 min. Samples were washed with 0.1% BSA/PBS and finally resuspended in 5 μl Lysis Buffer (0.1 mg/ml Proteinase K, 10 mM Tris-HCl pH 8.5 and 0.05% SDS) and lysed at 55°C for 3 h in a PCR cycler. 1.8% Triton X-100 with 5 mM PMSF was added to the cell lysate and incubated at RT for 10 min to quench the proteinase K followed by incubation at 55°C for 5 min to quench SDS in the reaction.

The DNA can be directly used for PCR amplification by adding 1 μL 25 μM i5 index primer, 1 μL 25 μM i7 index primer, 25 μl 2 x KAPA master mix and 17 μL 1 mM $MgCl_2$ to the 6 μL cell lysate for total 50 μl amplification condition. The reaction is set up by incubation at 72°C for 5 min, 98°C for 45 s, 14-18 cycles of (98°C for 15 s, 63°C for 30 s, 72°C for 1 min), and finally 72 °C extension for 5 min. After PCR, size selection was carried out by first 0.5 x SPRI beads to remove > 1 kb fragments, and second 0.4 x SPRI beads to the supernatant to obtain 200-1,000 bp fragments for sequencing. The libraries were sequenced with paired-end 150-bp reads on NovaSeq 6000 platform (Illumina).

Single-cell H3K27ac CoBATCH

The steps were the same as the Low input *in situ* ChIP until PAT binding. Cells were split into 96-well plates with 1,000-2,000 cells per tube in 80 μl Dig-Wash Buffer (with 0.05% Triton-X, cocktail, and 10 mM sodium butyrate) containing 0.83 μM PAT-T5 and 0.83 μM PAT-T7, and

incubated at 4°C for 1 h. Free PAT was then dislodged with 180 µL Dig-Wash Buffer containing 0.05% Triton X-100 three times at 5 min per time. The reaction was activated by suspending cells with 10 µL cold Reaction Buffer (10 mM TAPS-NaOH pH 8.3, 5 mM $MgCl_2$, 10% DMF, 0.05% Triton-X, 0.01% Digtonin and supplemented with cocktail, 10 mM sodium butyrate) in each tube. The reaction was gently mixed once after 30 min incubation and stopped by adding 8 µl 50 mM EDTA followed by further incubation at 4°C for 10 min after mixing well. Cells were combined and washed with 0.1% BSA/PBS three times, then passed through a 40 µm cell strainer to remove cell clumps. 20-25 cells were sorted into each well of a new 96-well plate which contains 3µL Lysis Buffer (0.1 mg/ml Proteinase K,10 mM Tris-HCl pH 8.5 and 0.05% SDS) and incubated at 55°C for 3 h in a PCR cycler.

Library enrichment was performed using two steps of PCR amplification as previously described(2). Briefly, the first step was performed by the addition of 0.5 µL 50 µM Truseq connector primer mix (Connector primer F:5′-ACACTCTTTCCCTACACGACGCTCTTCCGATCTTCGTCGGCAGCGTCTCCACGC-3′, Connector primer R:5′-GACTGGAGTTCAGACGTGTGCTCTTCCGATCTGTCTCGTGGGCTCGGCTGTCCCTGT-3′), 10 µL 5 × Q5 reaction buffer, 10 µL 5 × Q5 high GC enhancer, 1 µL 10 mM dNTP, 23.5 µL 1 mM MgCl2, 0.3 µL Q5 polymerase (NEB M0491S) to the 3 µL CoBATCH cell lysis in each tube and the reaction was set up by incubation at 72°C for 5 min, 95°C for 5 min, 13-16 cycles of amplification (95°C for 30 s, 63°C for 30 s, 72°C for 1 min), and final 72°C extension for 5 min. Then 0.5 µL 20 U/µl ExoI (NEB M0293S) was added and the reaction was incubated at 37°C for 30 min, and 72°C for 20 min. The second PCR enrichment was performed by the addition of 1 µl 10 mM Truseq index P5, 1 µl Truseq index P7, 2 µl 5 × Q5 reaction buffer, 2 µl 5 × Q5 high GC

enhancer, 0.5 μl 10 mM dNTP, 3.5 μl 1 mM MgCl2, 0.1 μl Q5 polymerase (NEB M0491S) to the mixture, and the reaction was set up by incubation at 95°C for 5 min, 5-7 cycles of amplification (95°C for 30 s, 63°C for 30 s, 72°C for 1 min), and final 72°C extension for 5 min. Finally, the PCR products were purified and selected by SPRI beads for 200-1000 bp.

Single-cell RNA-seq

Single-cell RNA-seq for sorted F4/80$^+$CD45$^+$7AAD$^-$ cells relied on the commercially available BMKGENE DG1000 System according to the manufacturer‘s instructions. In brief, 5000~8000 cells for each experimental condition were loaded to each channel. The cells were partitioned into Gel Beads in the instrument, where cell lysis and barcoded reverse transcription of RNA occurred. DNA enrichment and library construction were conducted. The resulting single-cell libraries were sequenced on a Novaseq 6000 platform.

Mouse heart collection and immunofluorescence staining

Postnatal mouse hearts were isolated at different time points after myocardial infarction and washed with PBS (10 mM KCl). The hearts were fixed with 4% paraformaldehyde overnight at 4°C, followed by washing with PBS 3 times at 5 min per time, 15% sucrose for 6 h and 30% sucrose overnight. Tissue was embedded in OCT medium at 4°C for 30 min and flash frozen in dry ice. The tissues were sectioned into 8 μm slices.

For immunofluorescence staining, slides were incubated at RT for 20 min before being washed with PBS twice and permeabilized with 0.1% Triton-X/PBS for 10 min. Then non-specific binding sites were saturated with 5% donkey serum in 0.1% Triton-X /PBS at RT for 1 h and the primary antibody was diluted in the same buffer and incubated overnight at 4°C in a Sealed box (anti-F4/80 (abcam, ab6640, 1:100); anti-S100A9 (R&D Systems, AF2065-SP, 1:200); anti-Cardiac Troponin I (abcam, ab56357, 1:200); anti-ARG1 (CST, 93668T, 1:200); anti-TNNI3 (abcam, ab56357,

1:200); anti-IL4 (Novus, NB100-64798, 1:50); anti-MHCII (Biolegend, 107615, 1:100), anti-KI67 (abcam, ab15580, 1:200); anti-PH3(CST, 9701s, 1:200); anti-CD31 (BD Biosciences, 553370, 1:200), anti-CXCR2 (Biolegend, 149603, 1:100)) (Supplemental Table 21). Primary antibodies were visualized by staining with Alexa-conjugated secondary antibodies: Alexa Fluor 488 donkey anti-goat (Invitrogen, A32816), Alexa Fluor 555 donkey anti-rabbit (Invitrogen, A31572) and Alexa Fluor 488 donkey anti-rabbit (Invitrogen, A32790) in 5% Donkey serum with 0.01% Triton-X /PBS at RT for 4 h. Nuclei were stained with DAPI and slides were imaged on the FV3000 Confocal Laser Scanning Microscope at 20× or 40× with immersion oil.

Masson trichrome staining and quantification

Postnatal mouse hearts were isolated at different time points after myocardial infarction and washed with PBS (10 mM KCl). The hearts were fixed in 4% paraformaldehyde at room temperature for 72 h, embedded in paraffin, and sectioned at 5 μm intervals. For trichrome staining, samples were sectioned at different levels below the suture (for MI hearts) or comparable levels (for Sham hearts), and stained using Masson's trichrome staining method as previously described (1). Briefly, sections were deparaffinized in xylene and rehydrated through the graded ethanol series (100%, 90%, 80%, 70%). The slides were then stained in ponceau acid fuchsin buffer for 10-15 minutes, 1% phosphomolybdic acid solution for 15 s and aniline blue solution for 2-3 minutes. 1% acetic acid solution was used for washing before changing the staining solution. The slides were dehydrated very quickly through 80%, 90%, and 100% gradient alcohol. Slides were mounted with xylene-containing mounting medium and imaged on Olympus FV1000 Confocal Microscope. The percentage of the infarcted LV wall was calculated by MIQuant (3).

H3K27ac CoBATCH data processing

Raw sequencing data were de-multiplexed to generate single cells with barcode combinations by custom scripts. Sequencing adapters were trimmed and the reads with low quality were filtered by Cutadapt (3.4). Clean reads were aligned to the mm10 reference genome using Bowtie2 (4) (2.2.5). Samtools (5) (1.13) was used to filter reads with mapping quality < 20, and Picard (2.26.1) (https://broadinstitute.github.io/picard/) was further utilized to remove duplicated reads. After removing the top-5% ranked cells with the highest deduplicated reads, single cells with deduplicated reads >1500 were kept for further analysis. The 24 pseudobulk groups were generated by aggregating single cells according to their sample information, and peaks were called by MACS2 (6) (2.2.7.1). CisTopic (7) (0.2.1) was applied to generate the single-cell peak-cell matrix using merged peaks from 24 groups.

Downstream analysis was mainly performed using Seurat (8) (4.1.1) and Signac (9) (1.7.0). The Seurat object was created using the peak-cell matrix, which only retains peaks showing signals in more than 10 cells. Graph-based clustering and non-linear dimension reduction were performed for visualization after removing the batch effect using Harmony (10). The peak-cell matrix was further converted into a gene activity matrix using the H3K27ac ChIP-seq signals at regions from 30 kb downstream of TTS and 50 kb upstream of TSS. Differential peaks and distinctly expressed genes in different clusters were assessed with the Seurat FindMarkers function.

Evaluation of the transcription factor activity from H3K27ac CoBATCH data

ChromVAR (11) was used to evaluate the activity of transcription factors. The position weight matrix was generated from the JASPAR2020 database. The cell-type-specific activities of transcription factors were calculated using the RunChromVAR function and differential activity was determined with the FindAllMarkers function (FDR < 0.05). Motif enrichment analysis was achieved with the FindMotif function.

Differential activity analysis of representative functional genes within subclusters

To evaluate the differences in enhancer activity of representative functional genes in MI versus Sham states within C4 and C6 clusters, each cluster was divided into 6 groups according to sampling time. 5 cells from each group were randomly selected with replacement, and repeated 50 times to obtain a new dataset of 1,500 cells for statistical analysis. The Kruskal-Wallis H test, followed by Dunn's test without *P*-value adjustment was used to calculate the significance of differences among groups.

Differential peak annotation within subclusters

The 8 pseudobulk groups were generated by aggregating single cells according to cluster information, and peaks were called by MACS2 (6). DiffBind (12) was then used to unify cluster peaks and re-quantify counts. The counts in each cluster were scaled by the total number of reads with edgeR (13) and were subsequently used to estimated differential peaks among clusters by fold change > 2. ChIPseeker (14) was applied to annotate cluster peaks with defining TSS region from -3kb to +3kb.

Generation of the cis-correlation networks (CCRN) of H3K27ac ChIP-seq signals

The interactions between H3K27ac peaks were predicted using the Cicero package (15) (1.3.6). The H3K27ac ChIP-seq library was converted into a cell dataset (CDS) using the as.cell_data_set function. The CDS objects were individually processed using the detect_genes function, and peaks with no signal were filtered out before converting to a Cicero CDS object. To generate cluster-specific cis-correlation networks (CCRN), we set a cutoff score of 0.2 and used a 100 kb genomic window.

GWAS SNP enrichment analysis

The SNPs associated with cardiovascular diseases were extracted from the GWAS database (16) (version1.0.2; retrieved on 2023-04-24), including the following keywords: heart failure, coronary artery disease, cardiotoxicity, cardiac embolism, congenital heart disease, cardiomyopathy, hypertension, AL amyloidosis, myocardial ischemia, hypotension, cardiac arrhythmia, stroke, heart disease, heart aneurysm, valve disease, septal defect and cor pulmonale. SNPs in LD ($r2 > 0.2$) were determined in PLINK (17) using European Individuals in 1000 Genomes (18) Data as the reference population (v3; retrieved from http://hgdownload.cse.ucsc.edu/gbdb/hg19/1000Genomes/phase3/). We then lifted all GWAS SNPs to the mouse genome using the UCSC utility liftOver (19).

To calculate the enrichment of GWAS traits within cell-type specific enhancer elements, a random background was constructed individually for each of the eight ChIP clusters. To achieve this, 1000 bp sites were randomly selected from the genome. The number of sites selected on each chromosome equaled the number of cluster-specific peaks for each ChIP cluster. This random selection was repeated 100 times and the number of GWAS SNPs within the random background sites was averaged across all 100 permutations. This random average was then compared to the observed number of SNPs within the cluster-specific enhancer regions determined by scChIP-seq. Statistical significance was evaluated via binomial test using the stat package in R (Binom.test) with option 'alternative = "greater"' (20).

K means and hierarchical clustering in the pheatmap (1.0.12) function were used to plot the H3K27ac ChIP cis-correlation networks. The cardiovascular diseases-related SNPs overlapped with C6-specific CCRN were selected to plot.

TF-TG network construction

To construct cluster-specific TF-TG networks, we selected the top 20 motifs in each cluster identified by ChromVAR (11). Cn_make_grn was used to predict TF-TG regulatory networks from CellNet (21) (0.1.1). Notably, the target genes were selected only if they were included in the top 50 cluster-specific genes sorted by the average value of log_2FC. In order to clarify the differences of TF regulatory networks between P1 and P10 among each cluster, the TF-TG networks were constructed in the P1 and P10 datasets separately according to the above method. For each cluster, the specific and shared TF-TGs between the two stages were distinguished by colors, and the network graph was generated separately by Cytoscape.

Milo for differential abundance analysis of cells within each cluster

Milo was used to perform the differential abundance analysis as previously described (22). Briefly, Milo objects were constructed from the SingleCellExperiment objects that were converted from original Seurat objects. K-nearest neighbor (KNN) graphs of cells in the whole dataset were then computed using the buildGraph function, based on the 20 leading dimensions corrected by Harmony. The representative subsets of neighborhoods that span the entire KNN graph was further defined through random sampling, with the neighborhood of 5 cells in scChIP-seq dataset or 70 cells in scRNA-seq dataset.

Next, the number of cells belonging to each experimental sample in individual neighborhoods was counted using the countCells function. Cells were randomly assigned to three batches using randomizr package in R to generate three biological replicates in the scRNA-seq dataset. After calculating the distances between nearest neighbors by calcNhoodDistance function, we performed differential analysis using testNhoods function and calculated a fold-change and adjusted *P*-value for each neighborhood. Furthermore, the identity of each neighborhood was defined by the most

abundant cell types. The threshold for significance of the false discovery rate (FDR) was set at 0.5 for the scChIP-seq dataset and 0.1 for the scRNA-seq dataset.

To evaluate the significance of difference in cell abundance between SB225002 and Vehicle treated groups in *S100a9*$^+$*Ly6c*$^+$ C7 and *Arg1*$^+$ C9 scRNA-seq clusters. Each cluster was divided into 2 groups according to sampling time. 500 cells from each group were randomly selected without replacement, and repeated 30 times to obtain a new dataset of 30,000 cells for statistical analysis. The P-value was determined by the Wilcox test was used to calculate the significance of differences among groups.

Differentially biased genes analysis along *Arg1*$^+$ fate or *S100a9*$^+$ fate

DeSeq2 (23) (1.40.1) was used to perform differential analysis (P-value<0.05 and Log$_2$FC>0.55) of genes biased along *Arg1*$^+$ fate or *S100a9*$^+$ fate.

Integration analysis of P1/P10 and adult H3K27ac CoBATCH datasets

To integrate the P1P10 and adult scChIP-seq datasets, peaks from two datasets were merged by reduce approach in the UnifyPeaks function. Subsequently, the count matrix was re-quantified based on cell fragments and merged peaks via the FeatureMatrix function. Top 2,000 highly variable peaks were selected from the adult dataset and used to construct anchors between the two datasets by FindTransferAnchors function. Finally, the TransferData function was applied to transfer P1P10 cell identities to adult cells according to linked anchors.

Enhancer activity comparison among P1, P10 and adult cells

To assess the enhancer activity of genes involved in wound healing and positive regulation of inflammation across P1, P10 and adult stages, we normalized the gene activity for each cell by the total H3K27ac ChIP-seq signals, multiplies this by a scale factor of 10000. To enable comparison

of enhancer activity changes across the three stages on the same scale, the normalized expression levels of genes were further scaled across the three stages.

Downstream analysis of scRNA-Seq data

The count matrices from P10-Sham_Vehicle, P10-MI_Vehicle, and P10-MI_SB225002 were generated from BMKGENE DG1000 data and then merged. The scRNA-seq data matrix was further processed with the Seurat pipeline (8) (4.1.1). For quality control, cells with a proportion of mitochondrial genes less than 10%, unique gene counts between 500 to 30,000, and detected genes between 500 to 5,000 were kept. The filtered count matrix was then normalized and scaled with the default pipeline. Principal component analysis (PCA) was performed based on the top 2,000 highly variable genes. The standard harmony integration pipeline was used to remove batch effects of three experimental groups. The first 20 integrated principal components were selected as input for UMAP reduction and unsupervised clustering. For the clustering of the whole dataset, the resolution was set to 0.47. MPC subsets were selected to be reprocessed with the first 10 principal components and the resolution was set to 0.8.

To identify the DEGs (differentially expressed genes) between P10-MI_ SB225002 and P10-MI_Vehicle groups, the FindAllMarkers function with default parameters was used on the normalized RNA data. The genes with average $\log_2 FC > 0.5$ were defined as DEGs in respective groups. Metascape database (v3.5.20230501) was used to perform Gene ontology analysis (24) and the DAVID database was used to enrich the KEGG pathway of the DEGs (25, 26).

To integrate of scChIP-seq and scRNA-Seq datasets, we utilized the Canonical correlation analysis (CCA) in the Seurat integrative pipeline (27). To achieve this, we applied the Seurat function FindTransferAnchors with default parameters on the CCA space to capture the shared feature between scChIP-seq and scRNA-Seq datasets. The first 20 PCA components of scRNA-

seq modality were used to calculate anchors, and Seurat's TransferData function was applied to assign each cell profiled by scChIP-seq to the cell type annotated by scRNA-Seq.

PCA analysis

To evaluate the similarities of single cells from RNA_C7 and RNA_C9 clusters under P10-Sham_Vehicle, P10-MI_Vehicle, and P10-MI_SB225002 conditions, C7 and C9 clusters were extracted from the original Seurat object and re-normalized. To separate each cell, the first 2000 high variability genes were selected through the "vst" method in the FindVariable function. The prcomp function was used to perform principal component analysis, and the PC1 and PC2 dimensions were used for visualization.

SCENIC analysis

We used the SCENIC package to analyze the regulatory network in C7 and C9. The raw counts were filtered by keeping genes with a sum of expression $> 3 \times 0.01 \times$ cell numbers detected in at least 1% of the cells. After filtering the expression matrix, we used the GENIE3 method to infer potential TF targets and converted them into co-expression modules, which were subsequently used to generate regulons based on DNA-motif analysis using the RcisTarget package. AUCcell was used to score regulon activity in each cell. The specific regulons in the MI_V and MI_SB groups were obtained according to the regulon specific scores that were calculated based on the calRSS function, and the David database was used to enrich the KEGG pathway of specific regulons corresponding target genes.

Statistical information

The statistical analysis was performed on R and all values were shown as mean±SEM. The number of replicates, statistical tests used, and test results were described in the figure legends for each

experiment. For all statistical tests, the 0.05 *P* value was considered statistically significant. In all figures, NS is not significant; $*P < 0.05$; $**P < 0.01$; $***P < 0.001$.

Study approval

All animal procedures were conducted in accordance with the local regulations and approved by the Institutional Animal Care and Use Committee of Southern Medical University (SMUL2023045).

Data availability

The single-cell CoBATCH dataset in neonatal and adult mouse generated in this study have been deposited in the GEO database at accession code GSE225615 and GSE263798 (secure token: slohagccllcdhwb and ihapsccuppsddyn). The single-cell RNA-seq data are available in the GEO under accession number in GSE235275 (secure token: gxazmcuapnwzrip). All custom code used in this study is available from the corresponding author upon reasonable request. A Supporting Data Values file is available online as supplemental material.

Author contributions

SSA designed and conceived the study. MZF and YLZ performed the bioinformatic analyses. STJ, LHX, XL, and YFL conducted the experiments. SSA and MZF wrote the paper with input from all other authors. All authors participated in data discussion and interpretation.

Acknowledgments

We thank all members of the Ai laboratory for their critical comments on this manuscript, and Aibin He from Peking University for his helpful discussion and feedback. This study was supported by grants from the National Key R&D Program of China (2022YFA1106200 and

2021YFA1102700), the National Natural Science Foundation of China (82270307 and 32200660), the Natural Science Foundation of Guangdong Province (2024B1515020058 and 2022A1515011325), the Natural Science Foundation of Guangzhou City (202201011012), Young Talent Support Project of Guangzhou Association for Science and Technology, and Guangdong Provincial Key Laboratory of Bone and Joint Degeneration Diseases.

References

1. Ai S, Yu X, Li Y, Peng Y, Li C, Yue Y, et al. Divergent Requirements for EZH1 in Heart Development Versus Regeneration. *Circulation research.* 2017;121(2):106-12.
2. Ai S, Xiong H, Li CC, Luo Y, Shi Q, Liu Y, et al. Profiling chromatin states using single-cell itChIP-seq. *Nat Cell Biol.* 2019;21(9):1164-72.
3. Nascimento DS, Valente M, Esteves T, de Pina Mde F, Guedes JG, Freire A, et al. MIQuant--semi-automation of infarct size assessment in models of cardiac ischemic injury. *PLoS One.* 2011;6(9):e25045.
4. Langmead B, and Salzberg SL. Fast gapped-read alignment with Bowtie 2. *Nat Methods.* 2012;9(4):357-9.
5. Danecek P, Bonfield JK, Liddle J, Marshall J, Ohan V, Pollard MO, et al. Twelve years of SAMtools and BCFtools. *Gigascience.* 2021;10(2).
6. Zhang Y, Liu T, Meyer CA, Eeckhoute J, Johnson DS, Bernstein BE, et al. Model-based analysis of ChIP-Seq (MACS). *Genome Biol.* 2008;9(9):R137.
7. Bravo Gonzalez-Blas C, Minnoye L, Papasokrati D, Aibar S, Hulselmans G, Christiaens V, et al. cisTopic: cis-regulatory topic modeling on single-cell ATAC-seq data. *Nat Methods.* 2019;16(5):397-400.
8. Hao Y, Hao S, Andersen-Nissen E, Mauck WM, 3rd, Zheng S, Butler A, et al. Integrated analysis of multimodal single-cell data. *Cell.* 2021;184(13):3573-87 e29.
9. Stuart T, Srivastava A, Madad S, Lareau CA, and Satija R. Single-cell chromatin state analysis with Signac. *Nat Methods.* 2021;18(11):1333-41.
10. Korsunsky I, Millard N, Fan J, Slowikowski K, Zhang F, Wei K, et al. Fast, sensitive and accurate integration of single-cell data with Harmony. *Nat Methods.* 2019;16(12):1289-96.
11. Schep AN, Wu B, Buenrostro JD, and Greenleaf WJ. chromVAR: inferring transcription-factor-associated accessibility from single-cell epigenomic data. *Nat Methods.* 2017;14(10):975-8.
12. Ross-Innes CS, Stark R, Teschendorff AE, Holmes KA, Ali HR, Dunning MJ, et al. Differential oestrogen receptor binding is associated with clinical outcome in breast cancer. *Nature.* 2012;481(7381):389-93.
13. Robinson MD, McCarthy DJ, and Smyth GK. edgeR: a Bioconductor package for differential expression analysis of digital gene expression data. *Bioinformatics.* 2010;26(1):139-40.

14. Wu DY, Bittencourt D, Stallcup MR, and Siegmund KD. Identifying differential transcription factor binding in ChIP-seq. *Front Genet.* 2015;6:169.
15. Pliner HA, Packer JS, McFaline-Figueroa JL, Cusanovich DA, Daza RM, Aghamirzaie D, et al. Cicero Predicts cis-Regulatory DNA Interactions from Single-Cell Chromatin Accessibility Data. *Molecular cell.* 2018;71(5):858-71 e8.
16. Buniello A, MacArthur JAL, Cerezo M, Harris LW, Hayhurst J, Malangone C, et al. The NHGRI-EBI GWAS Catalog of published genome-wide association studies, targeted arrays and summary statistics 2019. *Nucleic Acids Res.* 2019;47(D1):D1005-D12.
17. Purcell S, Neale B, Todd-Brown K, Thomas L, Ferreira MA, Bender D, et al. PLINK: a tool set for whole-genome association and population-based linkage analyses. *Am J Hum Genet.* 2007;81(3):559-75.
18. Genomes Project C, Auton A, Brooks LD, Durbin RM, Garrison EP, Kang HM, et al. A global reference for human genetic variation. *Nature.* 2015;526(7571):68-74.
19. Kuhn RM, Haussler D, and Kent WJ. The UCSC genome browser and associated tools. *Brief Bioinform.* 2013;14(2):144-61.
20. Ord T, Ounap K, Stolze LK, Aherrahrou R, Nurminen V, Toropainen A, et al. Single-Cell Epigenomics and Functional Fine-Mapping of Atherosclerosis GWAS Loci. *Circulation research.* 2021;129(2):240-58.
21. Cahan P, Li H, Morris SA, Lummertz da Rocha E, Daley GQ, and Collins JJ. CellNet: network biology applied to stem cell engineering. *Cell.* 2014;158(4):903-15.
22. Dann E, Henderson NC, Teichmann SA, Morgan MD, and Marioni JC. Differential abundance testing on single-cell data using k-nearest neighbor graphs. *Nat Biotechnol.* 2022;40(2):245-53.
23. Love MI, Huber W, and Anders S. Moderated estimation of fold change and dispersion for RNA-seq data with DESeq2. *Genome Biol.* 2014;15(12):550.
24. Zhou Y, Zhou B, Pache L, Chang M, Khodabakhshi AH, Tanaseichuk O, et al. Metascape provides a biologist-oriented resource for the analysis of systems-level datasets. *Nat Commun.* 2019;10(1):1523.
25. Huang da W, Sherman BT, and Lempicki RA. Systematic and integrative analysis of large gene lists using DAVID bioinformatics resources. *Nat Protoc.* 2009;4(1):44-57.
26. Sherman BT, Hao M, Qiu J, Jiao X, Baseler MW, Lane HC, et al. DAVID: a web server for functional enrichment analysis and functional annotation of gene lists (2021 update). *Nucleic Acids Res.* 2022;50(W1):W216-W21.
27. Stuart T, Butler A, Hoffman P, Hafemeister C, Papalexi E, Mauck WM, 3rd, et al. Comprehensive Integration of Single-Cell Data. *Cell.* 2019;177(7):1888-902 e21.
